# Chemical Constituents and Anticancer Activities of the Extracts from *Phlomis* × *commixta* Rech. f. (*P. cretica* × *P. lanata*)

**DOI:** 10.3390/ijms25020816

**Published:** 2024-01-09

**Authors:** Michalis K. Stefanakis, Olga St. Tsiftsoglou, Pavle Z. Mašković, Diamanto Lazari, Haralambos E. Katerinopoulos

**Affiliations:** 1 Department of Chemistry, University of Crete, Voutes, 71003 Heraklion, Greece; michstefanakis@yahoo.gr (M.K.S.); katerinc@uoc.gr (H.E.K.); 2 Laboratory of Pharmacognosy, Faculty of Health Sciences, School of Pharmacy, Aristotle University of Thessaloniki, 54124 Thessaloniki, Greece; olga@tsiftsoglou.gr; 3 Department of Food Technology, Faculty of Agronomy, University of Kragujevac, Cara Dušana 34, 32000 Čačak, Serbia; pavlem@kg.ac.rs

**Keywords:** *P.* × *commixta*, *Phlomis*, secondary metabolites, cytotoxic potent, phenolic derivatives, tyrosol esters

## Abstract

The present work is the first report on the ingredients of the *P.* × *commixta* hybrid, a plant of the genus *Phlomis*. So far, thirty substances have been isolated by various chromatographic techniques and identified by spectroscopic methods, such as UV/Vis, NMR, GC-MS and LC-MS. The compounds are classified as flavonoids: naringenin, eriodyctiol, eriodyctiol-7-O-*β*-D-glucoside, luteolin, luteolin-7-O-*β*-D-glucoside, apigenin, apigenin-7-O-*β*-D-glucoside, diosmetin-7-O-*β*-D-glucoside, quercetin, hesperetin and quercetin-3-O-*β*-D-glucoside; phenylpropanoids: martynoside, verbascoside, forsythoside B, echinacoside and allysonoside; chromene: 5,7-dihydroxychromone; phenolic acids: caffeic acid, *p*-hydroxybenzoic acid, chlorogenic acid, chlorogenic acid methyl ester, gallic acid, *p*-coumaric acid and vanillic acid; aliphatic hydrocarbon: docos-1-ene; steroids: brassicasterol and stigmasterol; a glucoside of allylic alcohol, 3-O-*β*-D-apiofuranosyl-(1→6)-O-*β*-D-glucopyranosyl-oct-1-ene-3-ol, was fully characterized as a natural product for the first time. Two tyrosol esters were also isolated: tyrosol lignocerate and tyrosol methyl ether palmitate, the latter one being isolated as a natural product for the first time. Moreover, the biological activities of the extracts from the different polarities of the roots, leaves and flowers were estimated for their cytotoxic potency. All root extracts tested showed a high cytotoxic activity against the Hep2c and RD cell lines.

## 1. Introduction

The genus *Phlomis* L. is one of the largest genera of the subfamily Lamioidae (Lamiaceae), which includes more than 100 species. Northwest Africa, Europe and Asia are the main places where this genus is found, but some species have been identified in southeastern Anatolia and northwestern Iraq [1]. Moench, in 1794, pointed out the morphological differences of the genus in two distinct genera, *Phlomis* and *Phlomioides*, and divided the genus into two groups distinguished by general differences, such as the chromosomal number (in the *Phlomis* group, it is generally 2n = 20, while in the *Phlomioides* group, it is 2n = 22) and the form (species of the *Phlomis* group are usually shrubs or subshrubs, while the *Phlomioides* group has mainly herbaceous plants) [2,3]. The flowers found in both groups are stalked and radially arranged by the axillary buds, while the lower edge of the flower is trilobate and the middle pod is wider than the other two. There are 12 records of the genus *Phlomis* in Europe [4], while in Greece, the genus is represented by 9 species. Three species of the genus are found in Crete: *Phlomis cretica* C. Preslin J. & C. Presl, *Phlomis fruticosa* L. and *Phlomis lanata* Willd. *Phlomis* is characterized as a difficult genus from a taxonomic point of view because it has a high frequency of hybridization and penetration between species [5]. The three species found on the island of Crete hybridize in pairs, producing three hybrids: *P.* × *cytherea* Rech. f. (*P. cretica* × *P. fruticosa*), *P.* × *commixta* Rech. f. (*P. cretica* × *P. lanata*) and *P.* × *sieberi* Vierh. (*P. fruticosa* × *P. lanata*). The hybrid *P.* × *commixta* has the intermediate morphological characteristics of *P. cretica* and *P. lanata*, as described above. The leaves are ovate–elliptic, with a sharply cut or rounded base. The hair on the leaves is dense or whitish. The bracts are linear or narrow lanceolate, with a length of 12–14 mm and a width of 1–3 mm. The width of the inflorescence vertebrae is 15–20 mm.

Over the last few years, there has been a rapid increase in the information available on the structures and pharmacological activities of new compounds isolated and identified from *Phlomis* species [6,7,8,9,10,11,12,13].

*Phlomis* species contain a diverse range of compounds, including iridoid glucosides [14,15,16], phenylpropanoid glycosides [17], flavonoid glycosides [18], lignans [19], neolignan glycosides [20], monoterpene glycosides [21], megastigmanes [22], diterpene glycosyl esters [23,24], nortriterpenes [25,26] and essential oils [5], which were reported to be responsible for various biological and pharmacological properties. Among them are the antinociceptive, anti-inflammatory [27,28,29], antioxidant [30,31,32,33,34], antimicrobial [35,36], wound-healing, antidiabetic [37,38] and antihemoroidal activity [39], as well as the antiplasmodial and anticancer activities [12,38,40,41,42,43].

To the best of our knowledge, no phytochemical investigation of the aerial parts and roots of *P.* × *commixta* has been previously carried out. The present work on the aerial and root system of the plant resulted in the isolation and identification of a series of components, including two new ones: 3-O-*β*-(3R)-D-apiofuranosyl-(1→6)-O-*β*-D-glucopyranosyl-(3S)-oct-1-en-3-ol (**1**) and the tyrosol methyl ether palmitate (**2**), as well as a third, tyrosol lignocerate (**3**), that is found for the first time in the genus *Phlomis* and the family Lamiaceae. The spectroscopic data of compound **3** are reported for the first time. The aim of this paper was to describe the isolation and structural elucidation of the components of the plant, with emphasis on the detailed structural identification of the new compounds, as well as the investigation of the anticancer potential of the plant’s extracts.

In the present study, the whole plant of the species *Phlomis* × *commixta* was collected, separated (leaves, flowers and roots), dried and extracted with solvents of increasing polarity, and the extracts were assessed for cytotoxicity in Hep2c and RD human cell lines, as well as in the L2OB cell line.

## 2. Results

### 2.1. Ιsolated Compounds

**Figure 1:**
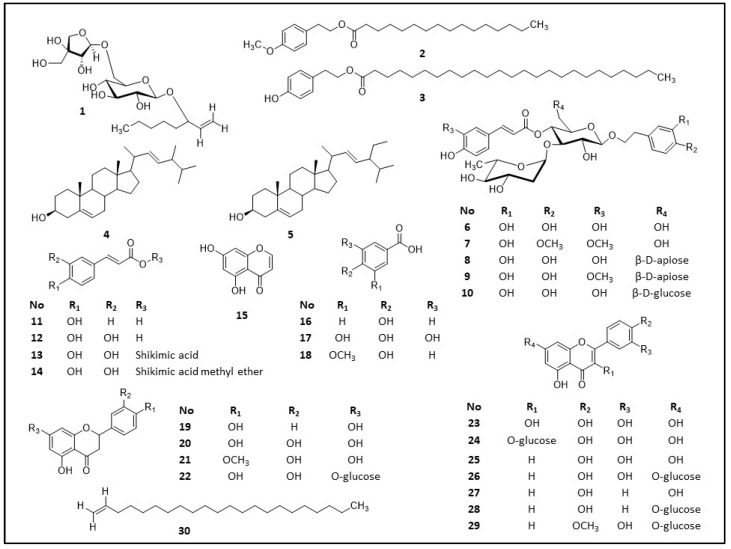
Isolated compounds from the leaves, flowers and roots of *Phlomis* × *commixta* Rech. f.

Various chromatographic techniques, such as column chromatography, pTLC and semipreparative HPLC, of the extracts from the leaves, flowers and roots of *Phlomis* × *commixta* Rech. F. led to the isolation of twenty-seven known compounds. Seven compounds were isolated from the roots (**2**, **3**, **4**, **5**, **6**, **8**, **9**), ten from the leaves (**1**, **6**, **8**, **10**, **18**, **21**, **23**, **24**, **26**, **27**) and sixteen from the flowers (**6**, **7**, **11**, **12**, **13**, **14**, **15**, **16**, **17**, **19**, **20**, **22**, **25**, **28**, **29**, **30**). It must be mentioned that compound (**6**) is present in all the examined parts of the plant. Also, compound (**8**) has been isolated from both the roots and leaves, but not from the flowers. Both compound (**6**) and (**8**) are phenylpropanoids, which suggests that the plant can biosynthesize these kinds of secondary metabolites in each plant part. The compounds were identified as brassicasterol (**4**) (see Supplementary Data Table S2) [44,45], stigasterol (**5**) (see Supplementary Data Table S3) [46,47], verbascoside (**6**) (see Supplementary Data Table S4) [48], martynoside (**7**) (see Supplementary Data Table S5) [48], forsythoside B (**8**) (see Supplementary Data Table S6) [49], allysonoside (**9**) (see Supplementary Data Table S7) [50], echinacoside (**10**) (see Supplementary Data Table S8) [51], p-coumaric acid (**11**) (see Supplementary Data Note S1) [52], caffeic acid (**12**) (see Supplementary Data Note S2) [53], chlorogenic acid (**13**) (see Supplementary Data Table S9) [54], chlorogenic acid methyl ester (**14**) (see Supplementary Data Table S10) [55], 5,7-dihydroxychromone (**15**) (see Supplementary Data Note S3) [56], p-hydroxybenzoic acid (**16**) (see Supplementary Data Table S11) [57], gallic acid (**17**) (see Supplementary Data Note S4) [58], vanillic acid (**18**) (See Supplementary Data Note S5) [57], naringenin (**19**) (see Supplementary Data Table S12) [59], eriodyctiol (**20**) (see Supplementary Data Note S6) [60], hesperetin (**21**) (see Supplementary Data Table S13) [61,62], eriodyctiol-7-O-*β*-D-glucoside (**22**) (see Supplementary Data Table S14) [63], quercetin (**23**) (see Supplementary Data Table S15) [64], isoquercetin (**24**) (see Supplementary Data Table S16) [57], luteolin (**25**) (see Supplementary Data Table S17) [65], luteolin-7-O-*β*-D-glucoside (**26**) (see Supplementary Data Table S18) [66], apigenin (**27**) (see Supplementary Data Table S19) [67,68], apigenin-7-O-*β*-D-glucoside (**28**) (see Supplementary Data Table S20) [69], diosmetin-7-O-*β*-D-glucoside (**29**) (see Supplementary Data Table S21) [70] and docos-1-ene (**30**) (see Supplementary Data Table S22) [71]. Figure 1 shows the structures of the isolates, which were determined using 1D and 2D NMR spectra and by comparing them to those reported in the literature.

### 2.2. Elucidation of the Compounds

**Figure 2:**
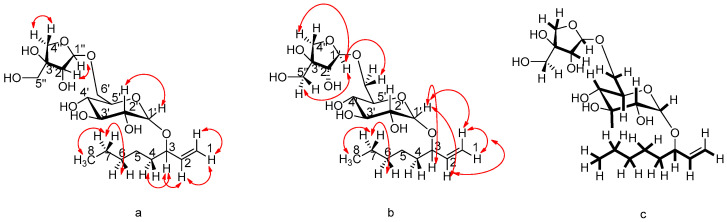
Main correlations of the COSY (**a**), NOESY (**b**) and TOCSY (**c**) (highlighted) of metabolite **1**.

This research led to the isolation of two new natural products: 3-O-*β*-(3R)-D-apiofuranosyl-(1→6)-O-*β*-D-glucopyranosyl-(3S)-oct-1-en-3-ol (**1**) and the tyrosol methyl ether palmitate (**2**). Moreover, this is the first study that fully describes the NMR of tyrosol lignocerate (**3**). The combination of NMR spectroscopic data with those of the high-resolution mass spectrum (HRMS) data led to the molecular formula C_19_H_34_O_10_, with a molecular ion at *m*/*z* = 445.2044 [M + HNa]^+^ (theor. C_19_H_34_NaO_10_; exact mass: 445.2050). At the same time, the mass spectrum (ESI-MS, negative ionization) showed, as the base peak, the fragment *m*/*z* = 421.4 [MH]^−^. Characteristic is the cleavage of the glycosidic O-bond, yielding a fragment of *m*/*z* = 131.0 of apiose, as well as a fragment of *m*/*z* = 289.3 [M-Apio-H]^−^. The next set of data were from the homonuclear 1D NMR and heteronuclear 2D NMR experiments (Figure 2). The most characteristic system in the proton spectrum of the compound is that of the geminal (vinyl) protons of the terminal double bond, which appear at *δ* 5.21 and 5.11 ppm (see Supplementary Data Figures S1 and S2). 

The HSQC spectrum confirmed the observation by correlation of these two protons to the secondary olefinic carbon at 116.3 ppm. The peak at 5.21 ppm appears as a ddd system, with a trans-vicinal coupling of *J*_1_ = 17.4 Hz, an allylic coupling of *J*_2_ = 1.7 Hz and a geminal coupling of *J*_3_ = 1.1 Hz. The peak at 5.11 ppm also appears as a ddd system, with a cis-vicinal coupling of *J*_1_ = 10.4 Hz, an allylic coupling of *J*_2_ = 1.7 Hz and a geminal coupling of *J*_3_ = 0.9 Hz. The C-2 olefinic carbon appears at 140.9 ppm, with the corresponding proton at 5.86 ppm. This proton also has the ddd system, with a trans-vicinal coupling of *J*_1_ = 17.4 Hz, a cis-vicinal coupling of *J*_2_ = 10.4 Hz and a value of *J*_3_ = 7.1 Hz, typical for the allylic proton. The high chemical shift *δ* of both the H-3 allylic proton and the C-3 tertiary allylic carbon (4.08 and 83.1 ppm, respectively) is indicative of the fact that the carbon is attached to a heteroatom; it is, in fact, attached to the epimeric oxygen of the glycoside, as will be shown below. The allylic proton is in a “key” position to identify the molecule, as it interacts through the HMBC spectrum with both the C-1 and C-2 olefinic carbons and the neighboring C-4 and C-5 aliphatic secondary carbons at *δ* 35.8 and 25.7 ppm, respectively (see Supplementary Data Table S1).

The glucoside bond between *β*-glucose and C-3 is doubly confirmed: (a) by the HMBC interaction between the anomeric proton H-1′ (*δ* 4.29 ppm) of glucose and C-3, and b) by the HMBC interaction of the H-3 (*δ* 4.08 ppm) of alcohol with the C-1 of glucose (*δ* 103.3).

The sequence of the carbons and the corresponding protons in the glucose molecule is clear, and can be confirmed by the data of the COSY, DEPT, HMBC, HSQC, NOESY and TOCSY spectra in, as well as by the coupling constant values of, the protons (*J*) in the proton spectrum, many of which are distinct (see Supplementary Data Figures S3–S9). A part of the data is in Table 1.

The peculiarity in the structure of apiose allows for the determination of the sequence of carbons and protons in the molecule. The anomeric proton H-1″ appears at *δ* 4.99 ppm and is correlated (HSQC) to the anomeric carbon that resonates at 110.8 ppm. 

The glucoside bond with glucose C-6′ is confirmed by (a) the NOESY interaction (see Supplementary Data Table S1) of the H-1″ of apiose with glucose H-6′ (3.56/3.92 ppm), (b) the interaction of H-1″ with C-6′ (68.4 ppm) in the HMBC spectrum and (c) the interaction of H-6′ glucose with the epimeric C-1″ carbon at δ 110.8 ppm in the same spectrum.

The apiose H-1″ epimeric proton gives a signal at 4.99 ppm, which splits into a doublet, with *J* = 2.5 Hz, due to the presence of the neighboring H-2″ proton, which appears at 3.88 ppm as a doublet, with *J* = 2.5 Hz. The carbon C-3″ at 80.6 ppm is easily recognized by ^13^C NMR, as it is the only quaternary carbon in the molecule, and therefore does not appear in the DEPT-135. The C4″ (75.0 ppm) is also easily recognizable. This carbon is secondary (DEPT-135) and has two protons at 3.75 and 3.95 ppm, which are diastereotopic, since C-3″ is a stereogenic center. These protons split only with each other, with a typical coupling constant of *J* = 9.6 Hz. The pack corresponding to C-5″, with a resonance at 65.7 ppm, carries two protons at 3.57 ppm that appear as a broad singlet in spite of the fact that the neighboring quaternary carbon C3″ is a stereogenic center. Mass spectrometry (ESI-MS) gave negative-ionization fragments, which can be interpreted by the fragmentation process analyzed in Figures S10 and S11 in Supplementary Material. The specific rotation value of the molecule was determined as [α]^D^_20_ = −420 (c = 2.0, CH_3_OH).

A literature review on this structure showed an initial report with spectroscopic data referring to the structure of 3-O-*β*-D-apiofuranosyl-(1→6)-O-*β*-D-glucopyranosyl-(3S)-oct-1-en-3-οl from the plant *Clerodendranthus spicatus* (Labiateae) [72].

**Figure 3:**
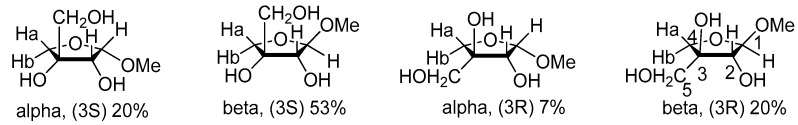
The four different diastereomers of apiose methyl glycosides.

Spectral data in this work were very similar to ours, with small differences in the ^13^C-NMR values for the apiose molecule. Based on their literature reference [73], our observations and the data on apiose methyl glycosides [74], we believe that the isolated compound is a 3-S apiose glycoside (Figure 3).

The correct apiose structure can be clarified based on the NOESY spectrum, showing the interactions of the protons in space. Indeed, H-1″ interacts strongly with H-5″ and H-4b″ protons, which are “facing down” in a “beta (3R)”-like structure.

This hypothesis was supported by the NOESY data, which further indicated the interaction of the H-5″ proton with the H-4b″ proton, as well as the absence of an interaction between the H-4a″ and H-1″ protons, which are “on opposite sides of the molecule” in a beta (3R) structure. It is, therefore, very likely that the correct structure of the molecule isolated from our group is 3-O-*β*-(3R)-D-apiofuranosyl-(1→6)-O-*β*-D-glucopyranosyl-(3S)-oct-1-en-3-ol. The small difference in the specific rotation of the compound, ${\left[a\right]}_{20}^{D}$ = −420, in relation to that of the literature, ${\left[\alpha \right]}_{20}^{D}$ = −340, is probably expected, given the small difference in the structure of the two molecules. 

Metabolite **2**: a tyrosol methyl ether palmitate (4-Methoxyphenethyl palmitate). The combination of the NMR and high-resolution mass spectrum (HRMS) spectroscopic data led to the molecular formula C_25_H_42_O_3_, with a molecular ion of *m*/*z* = 413.2666 [M + HNa]^+^ (theor. C_25_H_42_NaO_3_; exact mass: 413.3026) (see Supplementary Data Figures S12–S18). At the same time, the mass spectrum (ESI-MS, positive ionization) confirmed the molecular ion of *m*/*z* = 413.6 [M + HNa]^+^.

In the low-field range, two pairs of orthoconjugated aromatic protons, with peaks at *δ* 7.08 (*J* = 8.6 Hz, H-2/H-6) and 6.76 (*J* = 8.6 Hz, H-3/H-5), typical of an AΒ system, indicate the presence of a 1,4-substituted aromatic ring. There is also a triplet at 4.23 ppm (*J* = 7.0 Hz) for the ether moiety carbon (C-8) that split due to the coupling with the neighboring C-7 benzyl hydrogens, which resonate at 2.86 ppm, with a coupling constant of *J* = 7.0 Hz. Characteristic peaks in the spectrum are the singlet corresponding to the tyrosol methyl ether (C-9) at *δ* 3.48 and the triplet at *δ* 2.27, assigned to the methyne protons of the C-2′, split by the neighboring protons (H-3′) of the side chain, with a coupling constant of *J* = 7.3 Hz. A broad singlet at *δ* 1.25 ppm integrates to 22 protons, corresponding to an 11-carbon part of the aliphatic chain, and the triplet at *δ* 0.87 ppm is assigned to the terminal methyl group of this chain, split by the neighboring (C-15′) protons, with a coupling constant of *J* = 7.2 Hz.

**Figure 4:**
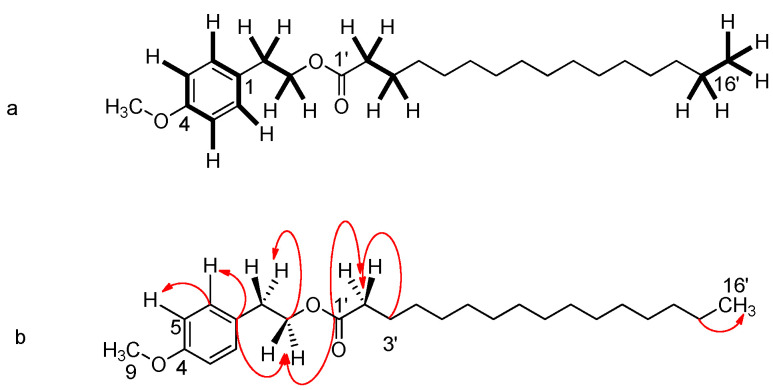
Main correlations of the COSY (highlighted) (**a**) and HMBC (**b**) of metabolite **2**.

In the carbon spectrum, the peak at 173.9 ppm confirmed the presence of the carbonyl carbon C-1′, and a very weak peak at 154.2 ppm was assigned to the quaternary C-4. By combining the NMR, HMBC (Figure 4) and HSQC spectroscopy techniques, it is understood that the peak at 115.2 ppm corresponds to C-3/C-5, since they interact only with the protons of the AB system, and the peak at 130.0 ppm corresponds to C-2/C-6, while the carbon at 50.9 ppm (C-9) corresponds to the methyl of tyrosol ether. The complete spectroscopic dataset for the compound is presented in Table 2 below. 

Metabolite **2** is reported for the first time as component of the hybrid or the genus *Phlomis*. The literature review on this structure showed a report by the Gargouri research team [75], reporting synthetic approaches to lipophilic ester derivatives of tyrosol. The NMR spectroscopic data of the synthetic compound were similar to those reported by our group. Therefore, to the best of our knowledge, metabolite **2** has been isolated as a plant component for the first time. The same group investigated the germicidal activity of tyrosol and synthetic esters against Gram (+) and Gram (−) bacteria. The minimum inhibitory concentration (MIC) against the microorganisms concerned, as well as their antileishmaniasis activity against *Leishmania major* and *L. infantum*, were examined.

Metabolite **3**: tyrosol lignocerate (2-(4′-hydroxyphenyl)-ethyl lignoceric ester), was identified using 1D and 2D NMR spectroscopy in combination with mass spectral data (ESI-MS), which showed a strong molecular ion of *m*/*z* = 487.4 [M-H]^−^ (see Supplementary Data Figures S19–S25). The signal sequence in the ^1^H-NMR spectrum showed several similarities to metabolite **2**, which indicated that it was an analogue of a tyrosol ester. The ^1^H-NMR spectrum showed two pairs of orthoconjugated aromatic protons, with low-field peaks at *δ* 7.07 (*J* = 8.5 Hz, H-2) and 6.76 (*J* = 8.5 Hz, H-3), typical of an AB system, indicating the presence of a 1,4-substituted aromatic ring. There is also a triplet at 4.23 ppm (*J* = 7.1 Hz) for the protons on the ether-type carbon (C-8), which are split due to the coupling with the adjacent benzylic hydrogens C-7 that resonate at 2.86 ppm, with a coupling constant of *J* = 7.1 Hz. Characteristic is also the triplet at *δ* 2.28, corresponding to its methylene proton C-2′, split by neighboring hydrogens (H-3′) of the side chain, with a coupling constant of *J* = 7.5 Hz. A broad singlet at *δ* 1.25 ppm integrates for 36 protons, corresponding to the presence of an aliphatic chain segment comprising 18 methylene moieties, and finally the triplet at *δ* 0.88 ppm corresponds to the terminal methyl of the aliphatic chain, split by the protons of the neighboring carbon (C-23′), with a coupling constant of *J* = 7.2 Hz. 

**Figure 5:**
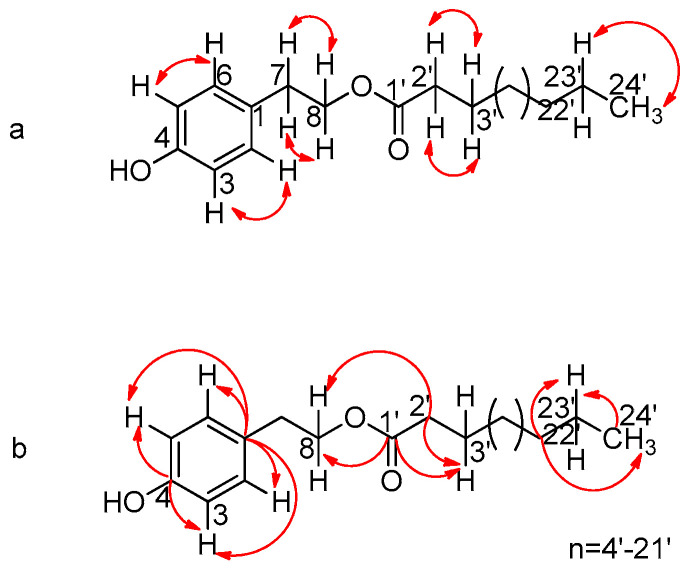
Key correlations of COSY (**a**) and HMBC (**b**) for metabolite **3**.

In the ^13^C NMR spectrum, the peak at 173.9 ppm confirmed the presence of the carbonyl carbon C-1′, whereas the peak at 154.2 ppm corresponds to the quaternary C-4. The combination of the NMR, HMBC (Figure 5) and HSQC techniques revealed that the peak at 115.2 ppm corresponds to C-3/C-5, since they interact only with the protons of the AB system. From the DEPT-135 range, it is clear that the secondary carbons of the aliphatic chain resonate in the range of 20–35 ppm, and the peak at 14.1 ppm corresponds to the terminal carbon of the chain. The complete spectroscopic data of the compound are presented in Table 3 below. 

Metabolite **3** was first isolated from the bark of the *Buddleja cordata* subsp. *cordata* of the family Loganiaceae [76]. It is isolated for the first time regarding both the hybrid and the genus *Phlomis* in general. This is the second time that this particular metabolite is mentioned in the literature. Compound **3**, according to the literature, showed moderate antibacterial activity against *Mycobacterium tuberculosis* (MIC = 64 μg/mL).

### 2.3. Cytotoxicity

The cytotoxic activity of the extracts from the roots, leaves and flowers of the hybrid *P*. × *commixta* was assessed using three different cell lines, i.e., Hep2c (human cervix carcinoma), RD (human rhabdomyosarcoma) and L2OB (murine fibroblast). There is a great interest regarding the biological activities of *Phlomis* spp. according to the literature. The results herein showed that all the samples from the roots expressed higher cytotoxic activity in the case of the Hep2c and RD cell lines, while the opposite was evidenced in the case of the L2OB cell line. The basic criterion for the cytotoxic activity of a plant extract is an IC50 < 30 µg/mL (Table 4).

## 3. Discussion

### 3.1. Chemotaxonomic Significance of the Secondary Metabolites

The genus *Phlomis* is well known for its richness in flavonoids and phenylpropanoid glycosides. A number of studies reveal that apigenin, luteolin, naringenin and their derivatives are very abundant in *Phlomis*’ polar extracts [12,25]. However, while there is only one report of the presence of diosmetin in *Phlomis* plants (*P. fruticosa*) [77], there is no one mentioning diosmetin-7-O-*β*-D-glucoside. It is noteworthy that quercetin is reported as a component only in three species of the genus *Phlomis* (*P. bracteosa* [78] and *P. elliptica* [79], *P. sterwartii* Hf. [38]). Zhang and Wang [80] initiated a study reporting the detection of phenolic acids in two Chinese *Phlomis* species. Phenolic acids isolated from *Phlomis* × *commixta* (caffeic acid, chlorogenic acid, p-coumaric acid, vanillic acid and gallic acid) have also been detected in acetone and methanol extracts of *P. umbrosa* and *P. megalantha*. There are very few references regarding the isolation and detection of caffeic acid (*P. stewartii* [7] and *P. lycunitis* [30], *P. armeniaca* [81]), p-hydroxy benzoic acid (*P. stewartii* [7] and *P. bracteosa* [78]) and chlorogenic acid (*P. olivieri* [82], *P. syriaca* [83]) in the genus *Phlomis*. The presence of chlorogenic acid and the methyl ester of an isomer (3-O-caffeoylquinic acid methyl ester) in *Phlomis* plants has been reported [25]; however, this is the first report on the presence of chlorogenic acid methyl ester in this genus.

Also, this is the first report of docos-1-ene (**30**) and 5,7-dihydroxy chromone (**15**) in the genus *Phlomis*. Stigmasterol is one of the most abundant sterols in the plant kingdom, and has also been isolated from the plants of the genus *Phlomis* (*P. cashmeriana* [8], *P. bracteosa* [84]). Brassicasterol, though, which is also a well-known sterol, is reported for the first time as component in the genus *Phlomis*. 

### 3.2. Anticancer Activity of the Extracts

Cancer is a disease that has a significant global impact on people. To treat and prevent this fatal condition, there is a continuous need for novel therapeutics. Natural substances are gaining attention from scientists because they are considered to have fewer hazardous side-effects than existing treatments, like chemotherapy. In order to create novel medications, secondary metabolites from the plant kingdom have been isolated and assessed for their anticancer properties.

There are many studies regarding the cytotoxic effects of the extracts from the aerial parts of various *Phlomis* species (Table 5). It must be mentioned that the current study provides the first report on the cytotoxic effects of the extracts from the roots of a *Phlomis* × *commixta* Rech. f. Strangely, our literature search did not reveal any reports concerning the evaluation of anticancer activity on the extracts derived from the roots of species belonging to the genus *Phlomis*. However, there are some studies on the isolation, characterization and antitumor activity of nortriterpenoids from the roots of *P. umbrosa* var *lutibracteata* [85], the antitumor activity of phlomiol extracted from the roots of *P. younghusbandii* [86,87] and phenylethanoid glycosides from the roots of *P. umbrosa* [88], as well as the chemical constituents in the roots of *P. medicinalis* and *P. younghusbandii* [23,89].

According to the bibliography, there is a great interest for the antitumor activity exhibited by the extracts of the species belonging to the genus *Phlomis.* Table 5 describes studies on the antitumor activity of the extracts from *Phlomis* spp. using in vitro techniques in various human cancer cell lines. In order to compile all this bibliographic data, we used free chemistry databases, such as PubChem, Scopus, Reaxys and Google Scholar. It is interesting that, in all these reports, the plant material was from the aerial or part of the aerial parts (such as the flowers or/and leaves).

According to our results, the plant part that exhibited the highest cytotoxicity was the roots. In terms of the basic criterion for the cytotoxic activity of a plant extract according to American National Cancer Institute (activity < 30 µg/mL), all polar solvent extracts from the roots showed to be active for the human cancer cell lines in doses between 19.61 and 29.77 µg/mL.

Another important observation is that, with the exception of the less-polar extracts from the leaves and the flowers of the plant, the rest of them show a moderate to strong antitumor effect, with IC50 values between 21.84 and 48.29 µg/mL. Moreover, the flowers’ extracts showed lower IC50 values than the leaves. Apart from that, the results show that none of the extracts derived after the liquid–liquid extraction of the methanolic extracts from the leaves had a stronger anticancer activity compared to the initial methanolic extracts. This result indicates the possible synergistic activity of the compounds extracted from the leaves. 

## 4. Materials and Methods

### 4.1. Plant Material

The plant material of *Phlomis* × *commixta* (Lamiaceae) was collected on 04/2012 from the village Tylisos of Heraklion, Crete, in Greece (coordinates: 35°18′42″ N, 25°01′07″ E). The plant was botanically identified by Prof. Dr. S. Pirintsos, Department of Biology, University of Crete, Greece, and a voucher specimen was deposited in the Department Herbarium. The plant samples were naturally dried (in the shade and in a well-ventilated environment), grinded by a laboratory mill (particle size approx. 1 mm) and stored in the darkness at room temperature.

### 4.2. Extraction and Isolation

The dried flower material (115 g) was extracted repeatedly in a Soxhlet apparatus (petroleum ether; dichloromethane; methanol). This process was thrice repeated (3 × 300 mL for 24 h each time), and after filtration and evaporation under reduced pressure, the petroleum extract (1.5 g), the dichloromethane extract (790 mg) and the methanol extract (27.0 g) residues were collected. The dried leaf herbal material (350 g) was extracted repeatedly at room temperature with petroleum ether, dichloromethane and methanol. This process was thrice repeated (3 × 300 mL for 24 h each time), and filtration and evaporation under reduced pressure afforded the petroleum extract (4.9 g), the dichloromethane extract (4.1 g) and the methanol extract (45.6 g) residues. The root systems of the plants were also extracted at room temperature with ethanol for six days under periodic shaking. The extract gave a solid residue weighing 7.2 g.

#### 4.2.1. Component Isolation from the Flowers

The flower methanol extract was evaporated under reduced pressure to yield a crude residue (29.6 g), which was then suspended in 1 L of boiling water and partitioned with Et_2_O, EtOAc and n-BuOH, respectively. The Et_2_O residue (3.1 g) was subjected to column chromatography (C.C.) on a Sephadex LH-20 (4.00 × 40.00 cm) and eluted with a mixture of CH_2_Cl_2_:MeOH (1:1) to obtain fractions A–Q. Fraction D (184.5 mg) was submitted to Sephadex LH-20 separation using a mixture of CH_2_Cl_2_:MeOH (1:2) as an eluent to obtain eight fractions (DA–DS). One of these fractions, DO (4.3 mg), was identified as naringenin. Fraction E (111.2 mg) was submitted to Sephadex LH-20 separation using a mixture of CH_2_Cl_2_:MeOH (1:2) as an eluent to obtain eight fractions (EA–ES). One of these fractions, EM (10.1 mg), was identified again as naringenin. Fraction F (27.1 mg) was submitted to Sephadex LH-20 separation using MeOH as an eluent to obtain eight fractions (FA–FO). From these, fraction FB (5.8 mg) was identified as p-hydroxybenzoic acid and fraction FC (1.2 mg) as 5,7-dihydroxychromone. Fraction G (11.6 mg) was separated on the Sephadex LH-20 using MeOH as an eluent to obtain eight fractions (GA–GO). From these, fractions GH (2.5 mg) and GI (1.1 mg) were identified as naringenin. Fraction H (17.1 mg) was separated on the Sephadex LH-20 using MeOH as an eluent to obtain eight fractions (HA–HC). From these, fraction HC (0.7 mg) was identified as caffeic acid, while fractions HL (1.8 mg) and HM (1.0 mg) were identified as naringenin, fraction HO (1.7 mg) was identified as a mixture of two compounds (eriodyctiol and luteolin) and the HP (1.7 mg) fraction was identified as luteolin. Fraction I (7.8 mg) was submitted to Sephadex LH-20 separation using a mixture of CH_2_Cl_2_:MeOH (1:2) as an eluent to obtain eight fractions (IA–IH). From these, fraction IE (3.2 mg) was identified again as luteolin. Fraction K (24.0 mg) was submitted to Sephadex LH-20 separation using a mixture of CH_2_Cl_2_:MeOH (1:2) as an eluent to obtain eight fractions (KA–KQ). From these, fraction KL (7.5 mg) was identified as a mixture of two compounds (eriodyctiol and luteolin) and fraction KM (1.0 mg) was identified as luteolin. 

The EtOAc residue (2.5 g) was subjected to column chromatography (C.C.) on the Sephadex LH-20 (4.00 × 40.00 cm) and eluted with mixture of CH_2_Cl_2_:MeOH (1:1) to obtain fractions A–O. Fraction C (382.4 mg) was submitted to C.C. on silica gel using CH_2_Cl_2_–MeOH mixtures of increasing polarity as eluents to obtain twenty-three fractions (CA–CW). One of these fractions, CR (13.5 mg), was identified as martynoside. Fractions D (610.2 mg) and E (1.1 g) were subjected to further chromatographic separations, as described below. Fraction D (610.2 mg) was submitted to C.C. on silica gel using CH_2_Cl_2_–MeOH–H_2_O mixtures of increasing polarity as eluents to obtain twenty-five fractions (DA–DZ). The hexane extract of fraction DA contained pure docos-1-ene (3.5 mg). Fraction DO (27.9 mg) was further fractionated by semipreparative HPLC (MeOH:H_2_O, 3:2, 1.00 mL/min) to obtain diosmetin-7-O-*β*-D-glucoside (Rt = 78.56 min, 1.1 mg). Fraction DU was identified as verbascoside (eluted with CH_2_Cl_2_–MeOH–H_2_O, 75:25:2.5, 265.6 mg). Fraction E (1.1 g) was submitted to C.C. on silica gel using CH_2_Cl_2_–MeOH–H_2_O mixtures of increasing polarity as eluents to obtain twenty-four fractions (EA–EY). Fraction EK (85.8) mg was submitted to Sephadex LH-20 (Merck KGaA, Darmstadt, Germany) separation using MeOH (100%) as an eluent to obtain twelve fractions (EKA–EKM). Fraction EKE (46.0 mg) was further purified on cellulose pTLC, which led to the isolation of chlorogenic acid methyl ester (14.3 mg, Rf = 0.87 on 30% acetic acid). Fraction EO (30.7 mg) was further purified on cellulose pTLC to yield apigenin-7-O-*β*-D-glucoside (1.0 mg, Rf = 0.28 on 30% acetic acid) and eriodyctiol-7-O-*β*-D-glucoside (16.6 mg, Rf = 0.69 on 30% acetic acid). Fraction ER was identified as verbascoside (eluted with CH_2_Cl_2_–MeOH–H_2_O, 75:25:2.5, 556.6 mg). Fraction EW was identified as chlorogenic acid (eluted with CH_2_Cl_2_–MeOH–H_2_O, 60:40:4.0, 50.7 mg). 

The butanol residue (3.5 g) was subjected to column chromatography (C.C.) on the Sephadex LH-20 (4.00 × 44.00 cm) and eluted with MeOH (100%) to obtain the fractions A–L. Fraction H (341.0 mg) was submitted to Sephadex LH-20 fractionation using MeOH (100%) as an eluent to obtain eight fractions (HA–HO). Fraction HD (10.6 mg) was further fractionated by semipreparative HPLC (MeOH:H_2_O, 1:1, 1.0 mL/min), which allowed for the isolation of diosmetin-7-O-*β*-D-glucoside (Rt = 32.72 min, 4.3 mg). Fraction HE (41.6 mg) was further fractionated by semipreparative HPLC (MeOH:H_2_O, 1:1, 1.0 mL/min), which allowed for the isolation of chlorogenic acid (Rt = 10.94 min, 2.5 mg) and diosmetin-7-O-*β*-D-glucoside (Rt = 29.74 min, 9.8 mg). Fraction HF (63.2 mg) was further fractionated by semipreparative HPLC (MeOH:H_2_O, 1:1, 1.0 mL/min), which allowed for the isolation of chlorogenic acid (Rt = 9.51 min, 3.0 mg), gallic acid (Rt = 14.34 min, 1.0 mg) and p-coumaric acid (Rt = 23.8 min, 2.8 mg) compounds.

#### 4.2.2. Component Isolation from the Leaves

The methanol extract was evaporated under reduced pressure to yield a crude residue (45.6 g), which was then suspended in 1 L of boiling water and partitioned with Et_2_O, EtOAc and n-BuOH, respectively. The EtOAc residue (3.4 g) was subjected to column chromatography (C.C.) on the Sephadex LH-20 (4.00 × 60.00 cm) and eluted with MeOH (100%) to obtain fractions A–L. Fraction E (88.0 mg) was further fractionated by semipreparative HPLC (MeOH:H_2_O, 1:1, 1.00 mL/min), which allowed for the isolation of echinacoside (Rt = 15.49 min, 11.9 mg) and isoquercetin (Rt = 40.34 min, 13.7 mg). Fraction H (98.0 mg) was submitted to Sephadex LH-20 fractionation using MeOH (100%) as an eluent to obtain nine fractions (HA–HP). Fraction HI (4.0 mg) was identified as apigenin. Fraction I (138.0 mg) was submitted to Sephadex LH-20 fractionation using MeOH (100%) as an eluent to obtain eleven fractions (IA–IL), which allowed for the isolation of quercetin from fraction ID (3.0 mg). Fraction IH (15.0 mg) was further fractionated by semipreparative HPLC (MeOH:H_2_O, 1:1, 1.00 mL/min), which allowed for the isolation of hesperetin (Rt = 46.47 min, 1.2 mg) and vanillic acid (Rt = 66.37 min, 1.0 mg).

The butanol residue (17.8 g) was subjected to VLC over silica gel (10 × 7 cm) using P.E., MeOH and H_2_O as eluent mixtures of increasing polarity to yield a total of twenty fractions (A–U). Fraction G (1.72 g) was submitted to Sephadex LH-20 fractionation using MeOH (100%) as an eluent to obtain twenty-one fractions (GA–GV). Fraction GH (104.5 mg) was further fractionated by semipreparative HPLC (MeOH:H_2_O, 1:1, 1.00 mL/min), which allowed for the isolation of 3-O-*β*-D-apiofuranosyl-(1→6)-O-*β*-D-glucopyranosyl-oct-1-en-3-ol) (Rt = 46.38 min, 4.7 mg). A portion of 167.0 mg from fraction GL (973.0 mg) was further purified on silica pTLC with EtOAc:AcOH:H_2_O/120:30:60, yielding forsythoside B (34.6 mg, Rf = 0.31) and verbascoside (38.6 mg, Rf = 0.55). Fraction GS (25.3 mg) was further purified on cellulose pTLC, which led to the isolation of luteolin-7-O-*β*-D-glucoside (6.2 mg) (Rf = 0.34 on EtOAc:AcOH:H_2_O/120:30:60).

#### 4.2.3. Component Isolation from the Roots

The petroleum ether extract was evaporated under reduced pressure to yield a crude residue (1.3 g), which was then submitted to C.C. on silica gel using CH_2_Cl_2_–EtOAc–MeOH mixtures of increasing polarity as eluents to obtain twenty-one fractions (A–V). Fractions I (16.5 mg), K (92.7 mg) and M (110.7 mg) were subjected to further chromatographic separations, as described below. Fraction I was further purified on silica pTLC, which led to the isolation of the tyrosol methyl ether palmitate (3.0 mg) (Rf = 0.56, on DM:EtOAc, 8:2). Fraction K was further purified on semipreparative HPLC (Hex:EtOAc, 4:1, 1.00 mL/min), which allowed for the isolation of tyrosol lignocerate (Rt = 12.20 min, 6.0 mg) and brassicasterol (Rt = 25.97 min, 12.3 mg). Fraction M was further fractionated by semipreparative HPLC (Hex:EtOAc, 4:1, 1.00 mL/min), which allowed for the isolation of stigasterol (Rt = 23.65 min, 1.0 mg). The EtOAc residue (1.8 g) was subjected to column chromatography (C.C.) on the Sephadex LH-20 (4.00 × 40.00 cm) and eluted with a mixture of MeOH (100%) to obtain fractions A–U. Fraction M (78.6 mg) was further fractionated by semipreparative HPLC (MeOH:H_2_O, 1:1, 1.0 mL/min), which allowed for the isolation of forsythoside B (Rt = 15.02 min, 10.5 mg) and verbascoside (Rt = 17.47 min, 4.6 mg).

The butanol residue (1.9 g) was subjected to column chromatography (C.C.) on the Sephadex LH-20 (4.00 × 60.0 cm) and eluted with MeOH to obtain fractions A–N. Fractions E (587.4 mg), F (555.5 mg) and G (78.5 mg) were subjected to further chromatographic separations, as described below. A portion of 40.0 mg from fraction E was further fractionated by semipreparative HPLC (MeOH:H_2_O, 1:1, 1.00 mL/min), which allowed for the isolation of forsythoside B (Rt = 14.75 min, 9.5 mg) and a mixture of two compounds (allysonoside and forsythoside B) (Rt = 16.05 min, 1.6 mg). Fraction F (86.8 mg) was further fractionated by semipreparative HPLC (MeOH:H_2_O, 1:1, 1.00 mL/min), which allowed for the isolation of forsythoside B (Rt = 14.68 min, 40.6 mg), verbascoside (Rt = 16.93 min, 2.2 mg) and a mixture of allysonoside and forsythoside B (Rt = 17.27 min, 1.8 mg). A portion of 36.0 mg from fraction G (78.0 mg) was further fractionated by semipreparative HPLC (MeOH:H_2_O, 1:1, 1.00 mL/min), which allowed for the isolation of forsythoside B (Rt = 14.87 min, 8.0 mg) and verbascoside (Rt = 17.24 min, 1.1 mg).

### 4.3. Cytotoxic Activity of the Studied Extracts

A standard cytotoxic activity test was performed [113] using the MTT (3-[4,5-dimethylthiazol-2-yl]-2,5-diphenyl tetrazolium bromide) assay [114,115]. The following cell lines were used (cell lines were donated from the collection of cell lines of the Institute of Virology, Vaccines and Serums “Torlak”, Belgrade, Serbia): RD (substrate: MEM Eagle/10% FCS) (cell line derived from human rhabdomyosarcoma), Hep2c (medium: MEM Eagle/5% FCS) (cell line derived from human cervix carcinoma–HeLa derivative) and L2OB (medium: MEM Eagle/10% FCS) (cell line derived from murine fibroblast), against which the activity of the extracts was measured. The results were expressed as IC50 values (µg/mL), a threshold which was defined as the concentration of an agent inhibiting cell survival by 50% in comparison to a vehicle-treated control [116]. The experiment was conducted in accordance with the method described by Mašković et al., 2015 [116].

## 5. Conclusions

The work presented in this publication is the first report on the isolation and the identification of the components of the *Phlomis* × *commixta* hybrid, an endemic plant of Crete, Greece. The component analysis included extraction from the flowers, the leaves and the roots of the plant, the subsequent component isolation using various chromatographic techniques and identification by spectroscopic methods, such as UV/Vis, NMR, GC-MS and LC-MS.

A total of thirty components were isolated, including flavonoids, phenylethanoic glycosides, phenolic acids, tyrosol esters, steroids, a chromone and an aliphatic hydrocarbon.

This report includes a detailed structural elucidation of allylic alcohol glycoside 3-O-*β*-D-apiofuranosyl-(1→6)-O-*β*-D-glucopyranosyl-oct-1-ene-3-ol, which was fully characterized as a natural product for the first time, and two tyrosol esters: tyrosol lignocerate and the tyrosol methyl ether palmitate, the former one mentioned only once in the literature as a component in a different species, and the latter one isolated as a natural product for the first time.

All the root extracts tested showed a high cytotoxic activity against the Hep2c and RD cell lines, with the leaf and flower extracts showing a moderate, though distinct, activity.

## Figures and Tables

**Table 1:** NMR spectroscopic data of metabolite **1** (CD_3_OD, 500 MHz).

#	^13^C	HSQC	DEPT 135	*δ* ^1^H	Multiplicity (*J*, Hz)
**Allylic part**					
1	116.3	H-1a	CH_2_	5.21	ddd, *J*_1_ = 17.4, *J*_2_ = 1.7, *J*_3_ = 1.1 Hz, 1H
		H-1b		5.11	ddd, *J*_1_ = 10.4, *J*_2_ = 1.7, *J*_3_ = 0.9 Hz, 1H
2	140.9	H-2	CH	5.86	ddd, *J*_1_ = 17.4, *J*_2_ = 10.4, *J*_3_ = 7.1 Hz, 1H
3	83.1	H-3	CH	4.08	dd, *J*_1_ = 13.1, *J*_2_ = 7.1 Hz, 1H
4	35.8	H-4a	CH_2_	1.51	m, 1H
		H-4b		1.68	dd, *J*_1_ = 10.2, *J*_2_ = 5.9 Hz, 1H
5	25.7	H-5a	CH_2_	1.39	m, 1H
		H-5b		1.32	m, 1H
6	33.1	H-6	CH_2_	1.31	m, 2H
7	23.7	H-7a H-7b	CH_2_	1.33	m, 1H
				1.37	m, 1H
8	14.4	H-8	CH_3_	0.90	t, *J* = 7.0 Hz, 3H
***β*-D-Glucose**					
1′	103.3	H-1′	CH	4.29	d, *J* = 7.8 Hz, 1H
2′	75.3	H-2′	CH	3.17	dd, *J*_1_ = 7.8, *J*_2_ = 4.2, 1H
3′	76.8	H-3′	CH	3.34	m, 1H
4′	71.7	H-4′	CH	3.25	dd, *J*_1_ = 8.7, *J*_2_ = 1.0 Hz, 1H
5′	78.2	H-5′	CH	3.33	m, 1H
6′	68.4	H-6a′	CH_2_	3.56	dd, *J*_1_ = 11.2, *J*_2_ = 5.2 Hz, 1H
		H-6b′		3.92	dd, *J*_1_ = 11.2, *J*_2_ = 1.8 Hz, 1H
***β*-D-Apiose**					
1″	110.8	H-1″	CH	4.99	d, *J* = 2.5 Hz, 1H
2″	78.0	H-2″	CH	3.88	d, *J* = 2.5 Hz, 1H
3″	80.6	H-3″	C	-	-
4″	75.0	H-4a″	CH_2_	3.95	d, *J* = 9.6 Hz, 1H
		H-4b″		3.75	d, *J* = 9.6 Hz, 1H
5″	65.7	H-5″	CH_2_	3.57	br.s, 2H

**Table 2:** NMR spectroscopic data of metabolite **2** (CDCl_3_, 500 ΜHz).

#	^13^C	HSQC	*δ* ^1^H	Multiplicity (*J*, Hz)	COSY	HMBC
1	130.1 *	-	-	-	-	H-2/H-6, H-7, H-8
2/6	130.0	H-2/H-6	7.08	d, *J* = 8.6 Hz, 2H	H-3/H-5	H-4, H-3/H-5
3/5	115.2	H-3/H-5	6.76	d, *J* = 8.6 Hz, 2H	H-2/H-6	-
4	154.2 *	-	-	-	-	H-2/H-6
7	34.3	H-7	2.86	t, *J* = 7.0 Hz, 2H	H-8	H-8
8	64.9	H-8	4.23	t, *J* = 7.0 Hz, 2H	H-7	H-7
9	50.9	H-9	3.48	s, 3H	-	-
1′	173.9	-	-	-	-	H-8, H-2′
2′	34.2	H-2′	2.27	t, *J* = 7.3 Hz, 2H	H-3′	-
3′	24.9	H-3′	1.60–1.57	m, 2H	H-2′	H-2′
4′/13′	29.7	H-4′/H-13′	1.25	br.s, 20H	-	-
14′	31.9	H-14′	1.25	br.s, 2H	-	H-16′
15′	22.6	H-15′	1.29–1.25	m, 2H	H-16′	H-16′
16′	14.1	H-16′	0.87	t, *J* = 7.2 Hz, 3H	H-15′	-

* Reported correlation are from the HMBC and HSQC spectra.

**Table 3:** NMR spectroscopic data of metabolite **3** (CDCl_3_, 500 ΜHz).

#	^13^C	HSQC	DEPT 135	*δ* ^1^H	Multiplicity (*J*, Hz)	COSY	HMBC
1	129.9	-	C	-	-	-	H-2/H-6 H-3/H-5
2/6	130.0	H-2/H-6	CH	7.07	d, *J* = 8.5 Hz, 2H	H-3/H-5	H-3/H-5 H-7, H-8
3/5	115.2	H-3/H-5	CH	6.76	d, *J* = 8.5 Hz, 2H	H-2/H-6	H-2/H-6
4	154.2	-	C		-	-	H-2/H-6 H-3/H-5
7	34.3	H-7	CH_2_	2.86	t, *J* = 7.1 Hz, 2H	H-8	H-2/H-6
8	64.9	H-8	CH_2_	4.23	t, *J* = 7.1 Hz, 2H	H-7	H-7
1′	173.9	-	C	-	-	-	H-8, H-2′, H-3′
2′	34.2	H-2′	CH_2_	2.28	t, *J* = 7.5 Hz, 2H	H-3′	H-8, H-3′
3′	24.9	H-3′	CH_2_	1.59	dt, *J*_1_ = 7.5, *J*_2_ = 7.0 Hz, 2H	H-2′	H-2′
4′/21′	29.6	H-4′/H-21′	(CH_2_)_18_	1.25	br.s, 36H	-	-
22′	31.9	H-22′	CH_2_	1.25	m, 2H	-	H-23′, H-24′
23′	22.6	H-23′	CH_2_	1.29	brq, *J* = 7.2 Hz, 2H	H-24′	H-24′
24′	14.1	H-24′	CH_3_	0.88	t, *J* = 7.2 Hz, 3H	H-23′	H-23′

**Table 4:** Cytotoxic activity of the extracts from *P.* × *commixta* in one murine (L2OB) and two human cell lines (Hep2c, RD) compared to the standard cytotoxic agent cis-diammine dichloroplatinum (cis-DDP).

Extract		IC50 Values (μg/mL)		
Solvent	Plant Parts	Hep2c Cells	RD Cells	L2OB Cells
Petroleum ether	roots	19.61 ± 0.39	23.18 ± 0.43	30.84 ± 0.31
Ethyl acetate	roots	20.74 ± 0.39	27.59 ± 0.29	33.76 ± 0.48
Butanol	roots	25.89 ± 0.21	28.57 ± 0.39	43.65 ± 0.74
Water	roots	26.32 ± 0.41	29.77 ± 0.18	46.74 ± 0.98
Petroleum ether	leaves	74.39 ± 0.12	98.38 ± 0.22	111.56 ± 0.37
Dichloromethane	leaves	36.53 ± 0.61	38.73 ± 0.77	54.76 ± 0.39
Methanol	leaves	37.88 ± 0.91	48.29 ± 0.45	62.72 ± 0.91
Petroleum ether (^a^ L.L.E of ^b^ ML)	leaves	52.92 ± 0.19	49.29 ± 0.33	85.38 ± 0.76
Ethyl acetate (L.L.E of ML)	leaves	52.78 ± 0.32	62.90 ± 0.28	88.75 ± 0.32
Butanol (L.L.E of ML)	leaves	79.61 ± 0.39	100.91 ± 0.48	121.42 ± 0.38
Water (L.L.E of ML)	leaves	91.44 ± 0.27	104.19 ± 0.64	129.39 ± 0.49
Petroleum ether	flowers	92.85 ± 0.24	108.29 ± 0.88	134.58 ± 0.29
Dichloromethane	flowers	29.47 ± 0.29	25.73 ± 0.93	32.46 ± 0.29
Methanol	flowers	38.52 ± 0.87	29.44 ± 0.31	35.30 ± 0.91
Butanol	flowers	21.84 ± 0.78	30.74 ± 0.28	45.21 ± 0.37
Water	flowers	38.63 ± 0.59	31.53 ± 0.98	48.93 ± 0.27
cis-DDP		0.94 ± 0.55	1.4 ± 0.97	0.72 ± 0.64
	American National Cancer Institute (NCI) < 30 μg/mL			

^a^ LLE: liquid–liquid extraction; ^b^ ML: methanol extract from leaves.

**Table 5:** Short synopsis of the cytotoxicity of the extracts from *Phlomis* spp. against human cancer cell lines.

*Phlomis* Species	Plant Parts	Cell Line	Bibliography
*P. olivieri*, *P. caucasica*, *P. anisodontea*, *P. bruguieri*, *P. kurdica*, *P. persica*		MCF-7, HepG2, HT29, A549 and MDBK	[90]
*P. lanceolata*	Total methanolic extract and partition fractions of flowering aerial parts	HT29, Caco2, T47D and NIH3T3	[91]
*P. syriaca*	Ethanolic extract from the flowers	MCF-7	[92]
*P. cypria* Post	70% aqueous methanol extract of herbal parts	SK-HEP-1 cancer cell line	[43]
*P. platystegia* Post	Aqueous extracts of aerial parts	HepG2	[93]
*P. linearis* Boiss. & Bal.	Aqueous extract	Mouse fibroblast L929 cell line, human H1299 cell line and human Caco-2 cell lines	[94]
	Hexane, diethyl ether, ethyl acetate and methanol extracts of the aerial parts	A549 and HT-29	[95]
*P. aurea* Decne and *P. floccosa* D		HEP-G2, HCT-116 and MCV-7	[96]
*P. aurea* Decne	Aerial parts extracts methylene chloride: methanol (1:1) and methanol: H_2_O (7:3)	CCRF–CEM leukemia cells	[97]
*P. persica* Boiss.	The aerial part of the plants macerated in ethanol (70%)	MCF-7 and MDA-MB231	[98]
*P. rigida* Labill.	Methanol extract from leaves and flowers	MCF-7, MDBK, HT-29 and A-549	[99]
*P. pungens* Willd.	Methanol extract of aerial parts powder	Caco-2, HepG2 and MCF-7 cell lines	[100]
*P. russeliana*	Ethanolic extract of aerial part	Caco-2 cell lines	[36]
	Leaf extract	HEK293 and MCF-7	[101]
*P. caucasica*	Aerial parts, 80% methanol extract	Human melanoma SKMEL-3 cells	[102]
*P. kurdica*			[103]
*P. persica*, *P. brugieri*, *P. olivieri*, *P. anisodontea*			[104]
*P. fruticosa* L.	Aerial parts, methanol extract	HGF-1, MCF-7, SiHa and HepG2	[105]
		A172 glioblastoma cell line	[106]
*P. sterwartii* Hf.	Leaves, flowers and whole plant	HepG2 cell lines	[38]
*P.* × *composita Pau*	Aerial parts	A549	[107]
*P. purpurea* L.	Flowering aerial parts		
*P. samia*	Aerial parts, methanol extract	HepG2 and MDA	[108]
*P. viscosa* Poiret	Crude ethanolic extracts from leaves	U-87 and MCF7	[109]
*P. thapsoides*	Aerial parts	Caco-2 and HepG2	[10]
*P. aucheri*	Aerial parts, methanol extract	PC-3, MCF-7, HepG2, CHO and B16-F10	[110]
*P. angustissima* and *P. fruticosa*	Methanol/water, ethyl acetate and water extracts of leaves and flowers	HeLa, MCF-7, ACC-201, OE-33 and HepG2	[111]
*P. bucharica* and *P. salicifolia*	Water, methanol, chloroform and hexane extracts from the whole plant	HeLa and HL-60 cell lines	[112]

## Data Availability

Data are contained within the article and Supplementary Materials.

## Supplementary Materials

The following supporting information can be downloaded at: https://www.mdpi.com/article/10.3390/ijms25020816/s1.
