# Supplementary material for: Chemical Constituents and Anticancer Activities of the Extracts from Phlomis × commixta Rech. f. (P. cretica × P. lanata)
Source: Int J Mol Sci. 2024 Jan 9;25(2):816. doi: 10.3390/ijms25020816 (PMC10815138; doi:10.3390/ijms25020816)
Supplement: Supplementary file 1 [file ijms-25-00816-s001.zip › ijms-2804280-supplementary.pdf]

Supporting Information

Chemical constituents and anti-cancer activities of the extracts from *Phlomis* × *commixta* Rech. f. (*P. cretica* × *P. lanata*)

Michalis K. Stefanakis [1], Olga S. Tsiftsoglou [2], Pavle Z. Mašković [3], Diamanto Lazari [2]* and Haralambos E. Katerinopoulos [1]

[1] Department of Chemistry, University of Crete, 71003, Voutes, Heraklion, Crete, Greece; michstefanakis@yahoo.gr (M.K.S); katerinc@uoc.gr (H.E.K)
[2] Laboratory of Pharmacognosy, Faculty of Health Sciences, School of Pharmacy, Aristotle University of Thessaloniki, 54124 Thessaloniki, Greece; olga@tsiftsoglou.gr (O.S.T); dlazari@pharm.auth.gr (D.L)
[3] Department of Food Technology, Faculty of Agronomy, University of Kragujevac, Cara Dušana 34, 32000 Čačak, Republic of Serbia; pavlemaskovic@yahoo.com (P.Z.M)

Table S1: NMR spectroscopic data of metabolite **(1)** 3-O-β-(3R)-D-apiofuranosyl- (1→6)-O-β-D-glucopyranosyl-(3S)-oct-1-en-3-ol, (CD_3OD, 500 MHz).

#	^{13}C	HSQC	DEPT 135	$\delta\,^1H$	Multiplicity, (J, Hz)	COSY	TOCSY	NOESY	HMBC
**Allylic part**									
1	116.3	H-1a	CH_2	5.21	ddd, J_1 = 17.4, J_2 = 1.7, J_3 = 1.1 Hz, 1H	H-1b, H-2	H-2, H-3, H-4a, H-4b, H-5a, H-5b, H-6, H-7a, H-7b	H-1b, H-2, H-3, H-4a, H-4b, H-5a, H-5b, H-1′	H-3
		H-1b		5.11	ddd, J_1 =10.4, J_2 = 1.7, J_3 = 0.9 Hz, 1H	H-1a, H-2	H-2, H-3, H-4a, H-4b, H-5a, H-5b, H-6, H-7a, H-7b	H-1a, H-2	
2	140.9	H-2	CH	5.86	ddd, J_1 = 17.4, J_2 = 10.4, J_3 = 7.1 Hz, 1H	H-1a, H-1b, H-3	H-1a, H-1b, H-3, H-4a, H-4b, H-5a, H-5b, H-6, H-7a, H-7b	H-1a, H-1b, H-3, H-1′	H-1a, H-3, H-4a, H-4b
3	83.1	H-3	CH	4.08	dd, J_1 = 13.1, J_2 = 7.1 Hz, 1H	H-2, H-4a, H-4b	H-1a, H-1b, H-2, H-4a, H-4b, H-5a, H-5b, H-6, H-7a, H-7b, H-8	H-1a, H-2, H-4a, H-4b, H-5a, H-5b, H-1′	H-1a, H-1b, H-2, H-4a, H-4b, H-1′
4	35.8	H-4a	CH_2	1.51	m, 1H	H-3, H-4b, H-5a	H-1a, H-1b, H-2, H-3, H-4b, H-5b, H-5b, H-6, H-7a, H-7b, H-8	H-2, H-3, H-4b, H-5b, H-1′	H-3
		H-4b		1.68	dd, J_1 =10.2, J_2 = 5.9 Hz, 1H	H-3, H-4a, H-5a, H-5b	H-1a, H-1b, H-2, H-3, H-4a, H-5a, H-5b, H-6, H-7a, H-7b, H-8	H-2, H-3, H-4a, H-5b, H-1′	
5	25.7	H-5a	CH_2	1.39	m, 1H	H-4a, H-4b	H-1a, H-1b,H-2, H-3, H-4a, H-4b, H-5b, H-6, H-7a, H-7b, H-8	H-3, H-4b, H-7	H-3
		H-5b		1.32	m, 1H	H-4b	H-1a, H-1b, H-2, H-3, H-4a, H-4b, H-5a, H-6, H-7a, H-7b, H-8	H-3, H-4a, H-4b	
6	33.1	H-6	CH2	1.31	m, 2H	H-5a	H-1a, H-1b, H-2, H-3, H-4a, H-4b, H-5a, H-5b, H-7a, H-7b, H-8	H-3, H-4a, H-4b	H-5b, H-7a, H-8

#	^{13}C	HSQC	DEPT 135	$\delta\,^1H$	Multiplicity, (*J*, Hz)	COSY	TOCSY	NOESY	HMBC
7	23.7	H-7a	CH_2	1.33	m, 1H	H-6, H-8	H-1a, H-1b, H-2, H-3, H-4a, H-4b, H-5a, H-5b, H-6, H-7b, H-8	H-7b, H-8	H-6, H-7a, H-8
		H-7b		1.37	m, 1H	H-6, H-8	H-1a, H-1b, H-2, H-3, H-4a, H-4b, H-5a, H-5b, H-6, H-7a, H-8	H-7a, H-8	
8	14.4	H-8	CH_3	0.90	t, J = 7.0 Hz, 3H	H-7a, H- 7b	H-3, H-4a, H-4b, H-5a, H-5b, H-6, H-7a, H-7b	H-7a, H-7b	H-7a
***β*-D-Glucose**									
1′	103.3	H-1′	CH	4.29	d, J = 7.8 Hz, 1H	H-2′	H-2′, H-3a′, H-3b′, H-4′, H-5′, H-6a′	H-3, H-2′, H-3′, H-4′	H-3, H-2′, H-3′, H-4a, H-4b, H-2
2′	75.3	H-2′	CH	3.17	dd, J_1 = 7.8, J_2= 4.2, 1H	H-1′	H-1′, H-3a′, H-3b′, H-4′, H-5′, H-6a′	H-1′, H-3a′, H-3b′	H-1′
3′	76.8	H-3′	CH	3.34	m, 1H	H-2′, H-4′	H-1′, H-2′, H-4′, H-5′, H-6a′, H-6b′	H-1′, H-6a′, H-6b′	H-1′, H-4′
4′	71.7	H-4′	CH	3.25	dd, J_1 = 8.7, J_2 = 1.0 Hz, 1H	H-5′	H-1′, H-2′, H-3a′, H-3b′, H-5′, H-6a′, H-6b′	H-1′, H-2′, H-6a′, H-6b′	H-3′, H-5′
5′	78.2	H-5′	CH	3.33	m, 1H	H-4′, H-6a′	H-1′, H-2′, H-3b′, H-3b′, H-4′, H-6a′, H-6b′	H-1′, H-6a′, H-6b′	H-2′, H-4a′, H-5′
6′	68.4	H-6a′	CH_2	3.56	dd, J_1 = 11.2, J_2 = 5.2 Hz, 1H	H-6b′	H-4′, H-5′, H-6b′	H-1″, H-6b′	H-4′
		H-6b′		3.92	dd, J_1 = 11.2, J_2 = 1.8 Hz, 1H	H-6a′, H-1″	H-5′	H-3′, H-5′, H-6a′, H-1″	H-1″
***β*-D-Apiose**									
1″	110.8	H-1″	CH	4.99	d, J = 2.5 Hz, 1H	H-2″	H-2″	H-5′, H-6a′, H-6b′,	H-6a′, H-6b′,

#	^{13}C	HSQC	DEPT 135	$\delta\,^1H$	Multiplicity, (*J*, Hz)	COSY	TOCSY	NOESY	HMBC
								H-2″, H-4b″, H-5″	H-2″, H-4b″
2″	78.0	H-2″	CH	3.88	d, *J* = 2.5 Hz, 1H	H-1″	H-1″	H-6a′, H-1″, H-4a″	H-4b″, H-5″
3″	80.6	H-3″	C	-	-	-	-	-	H-1″, H-4b″, H-5″
4″	75.0	H-4a″	CH_2	3.95	d, *J* = 9.6 Hz, 1H	H-4b″	H-4b″	H-6a′, H-1″, H-4b″, H-5″	H-1″
		H-4b″		3.75	d, *J* = 9.6 Hz, 1H	H-4a″	H-4a″	H-5″	H-5″
5″	65.7	H-5″	CH_2	3.57	br.s, 2H	-	-	H-4′, H-1″, H-4b″	H-2″, H-4a″

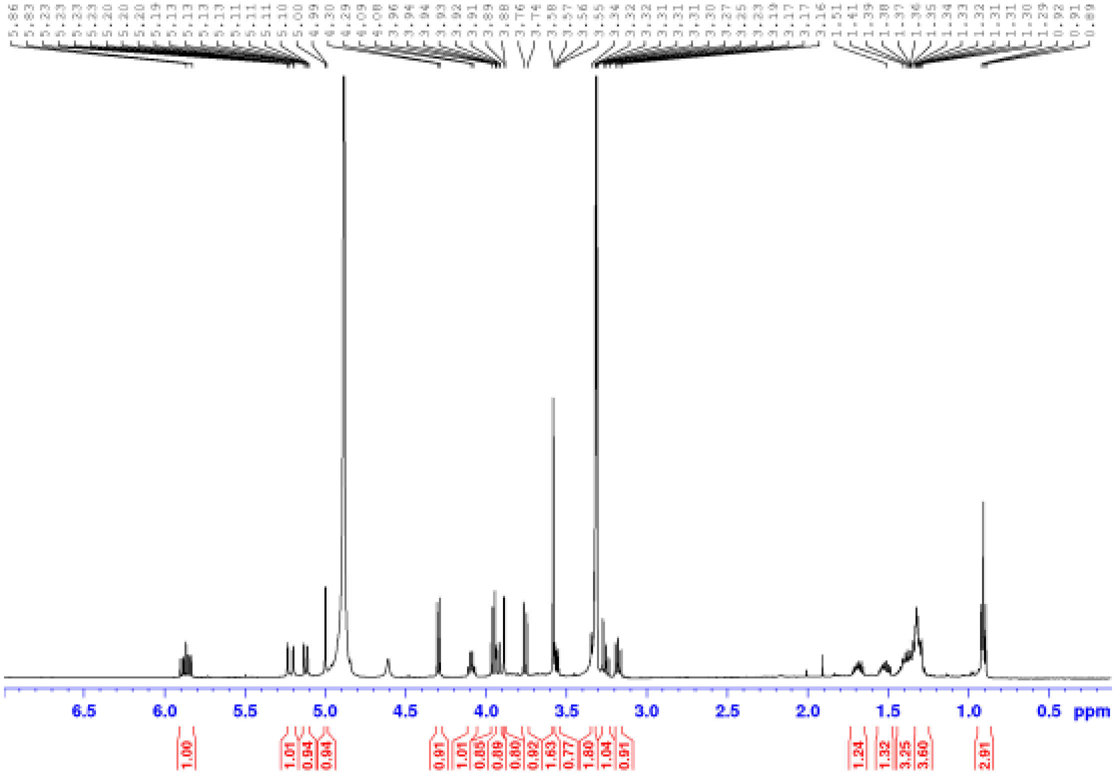

Figure S1: ^{1}H-NMR spectrum of compound **1** (CD_3OD, 500 MHz).

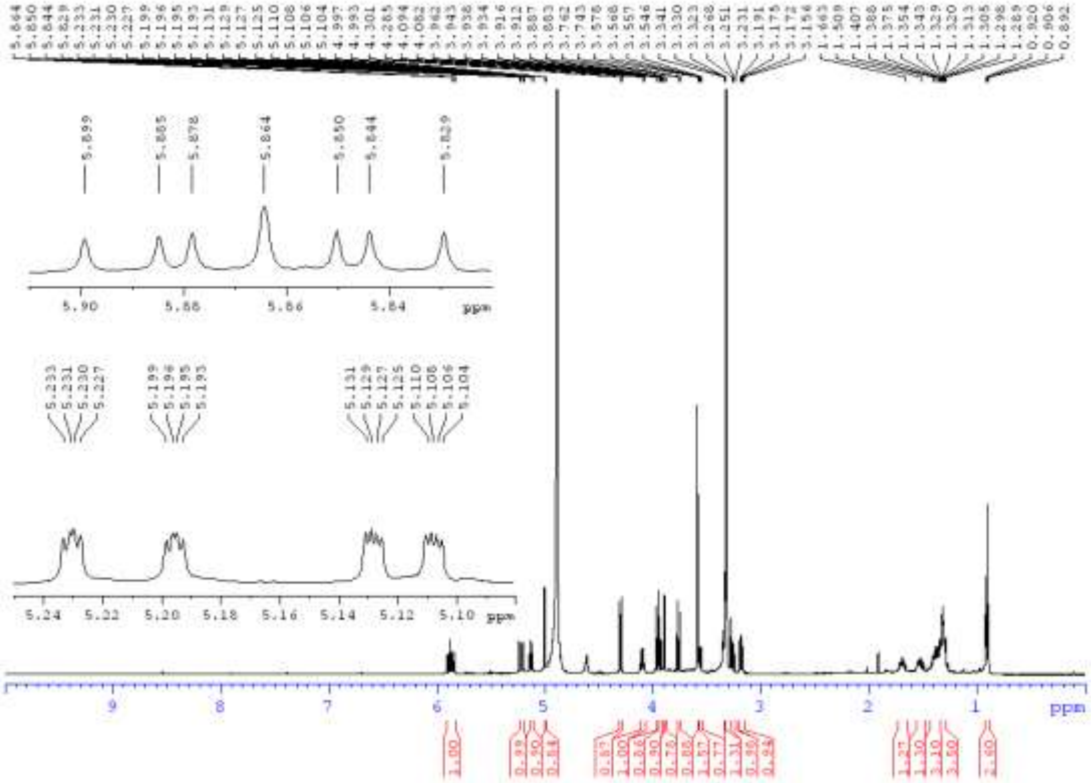

Figure S2: ^{1}H-NMR spectrum of the compound **1** (CD_3OD, 500 MHz).

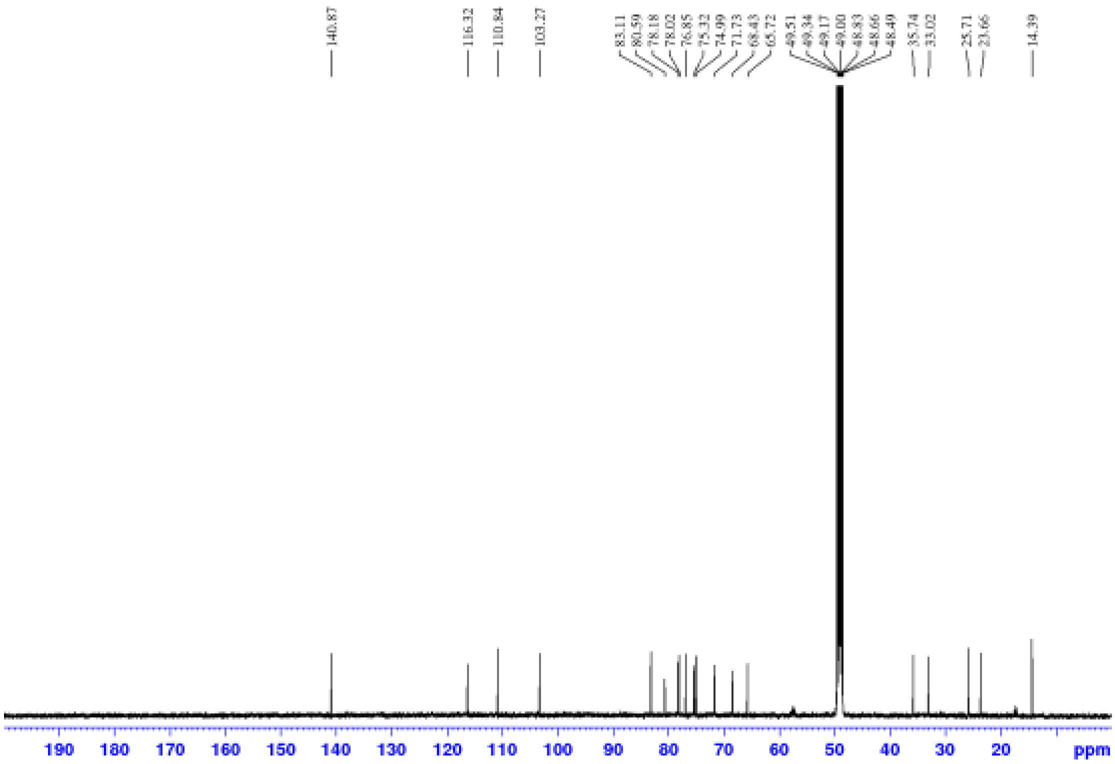

Figure S3: ^{13}C-NMR spectrum of compound **1** (CD_3OD, 125 MHz).

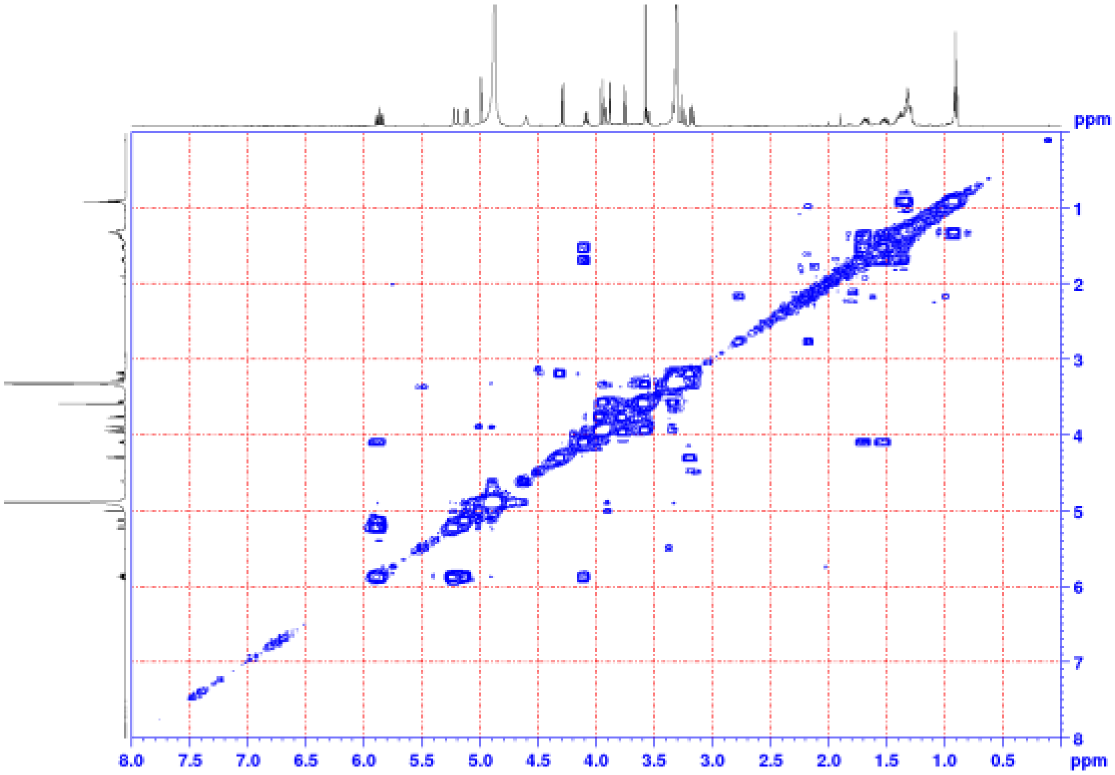

Figure S4: COSY spectrum of compound **1** (CD_3OD, 500 MHz).

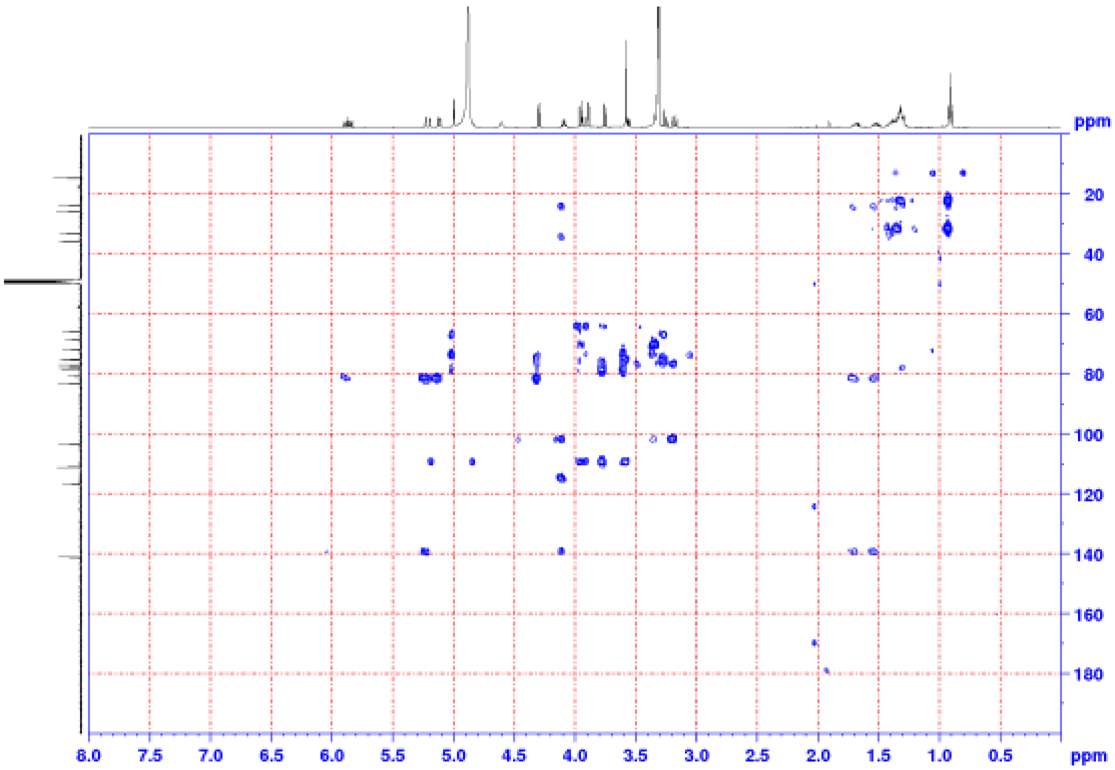

Figure S5: HMBC spectrum of compound **1** (CD_3OD, 500 MHz).

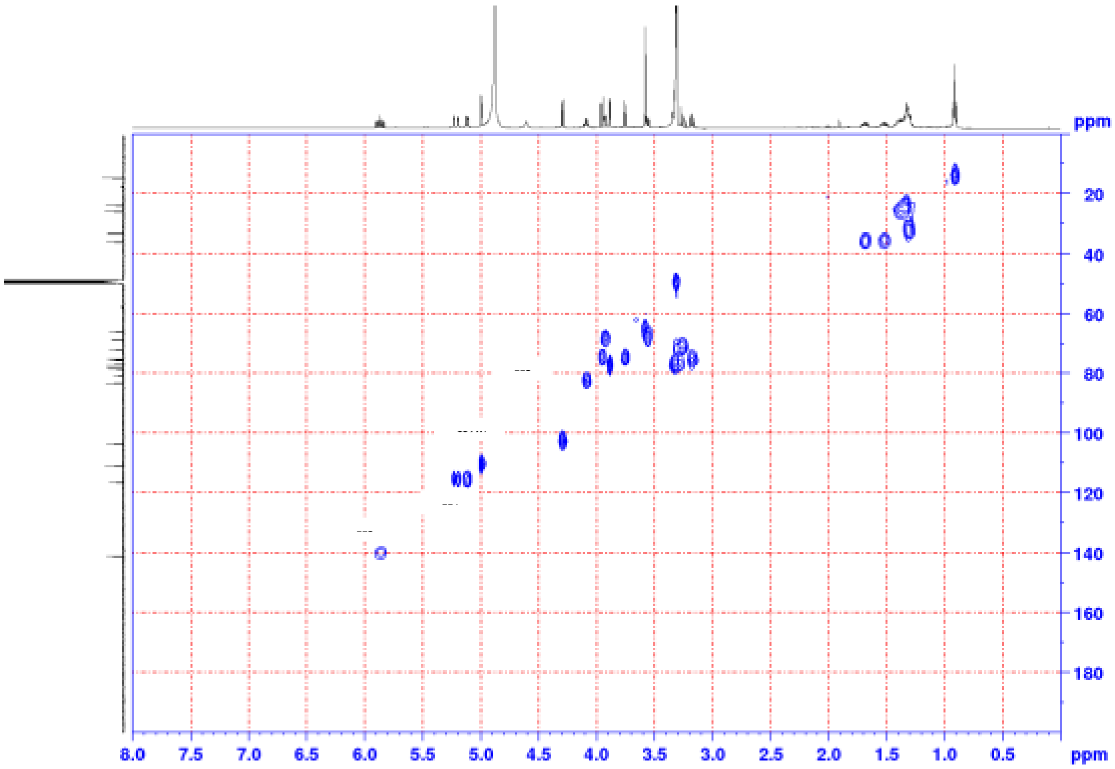

Figure S6: HSQC spectrum of compound **1** (CD_3OD, 500 MHz).

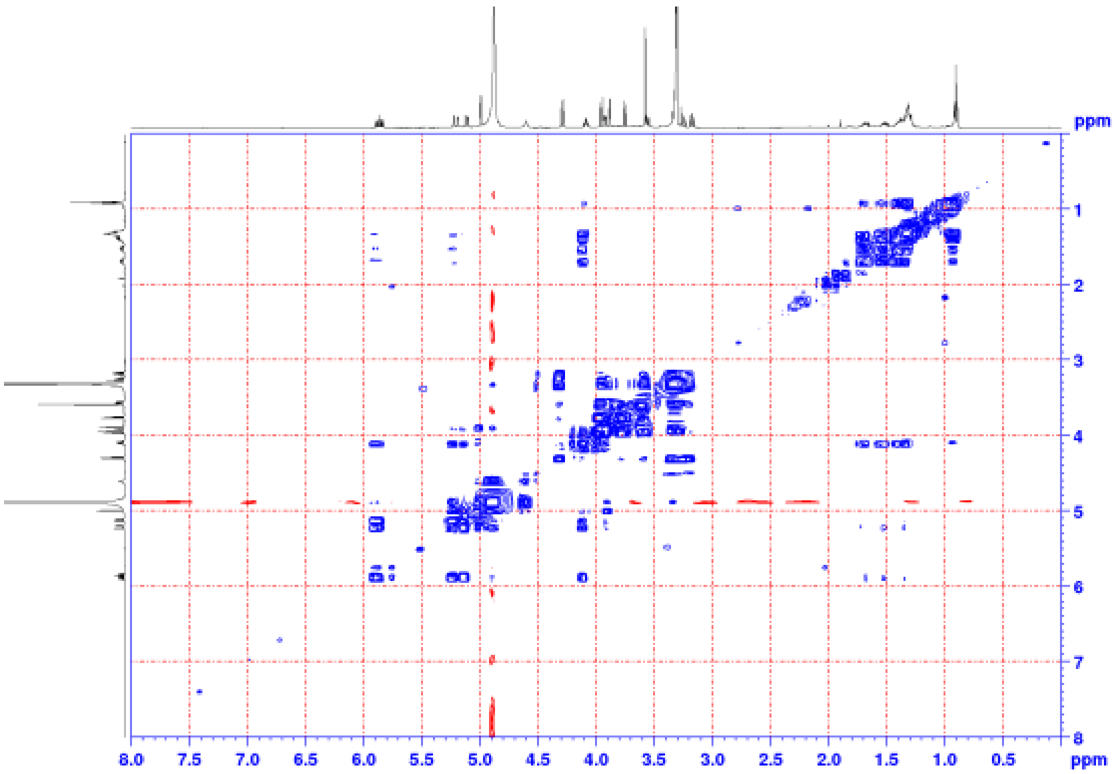

Figure S7: TOCSY spectrum of compound **1** (CD_3OD, 500 MHz).

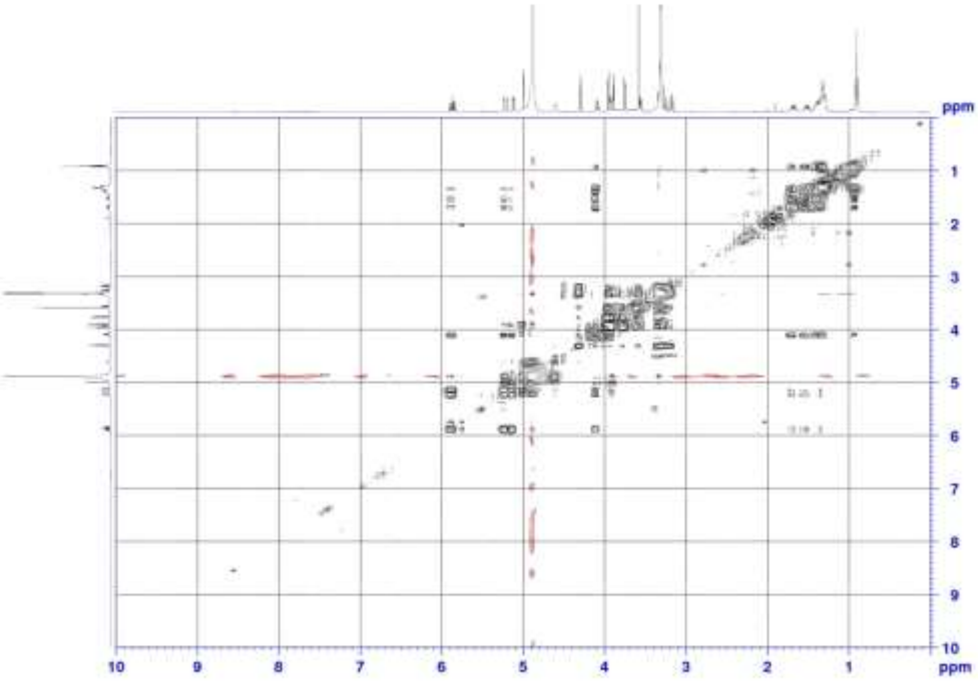

Figure S8: NOESY spectrum of compound **1** (CD_3OD, 500 MHz).

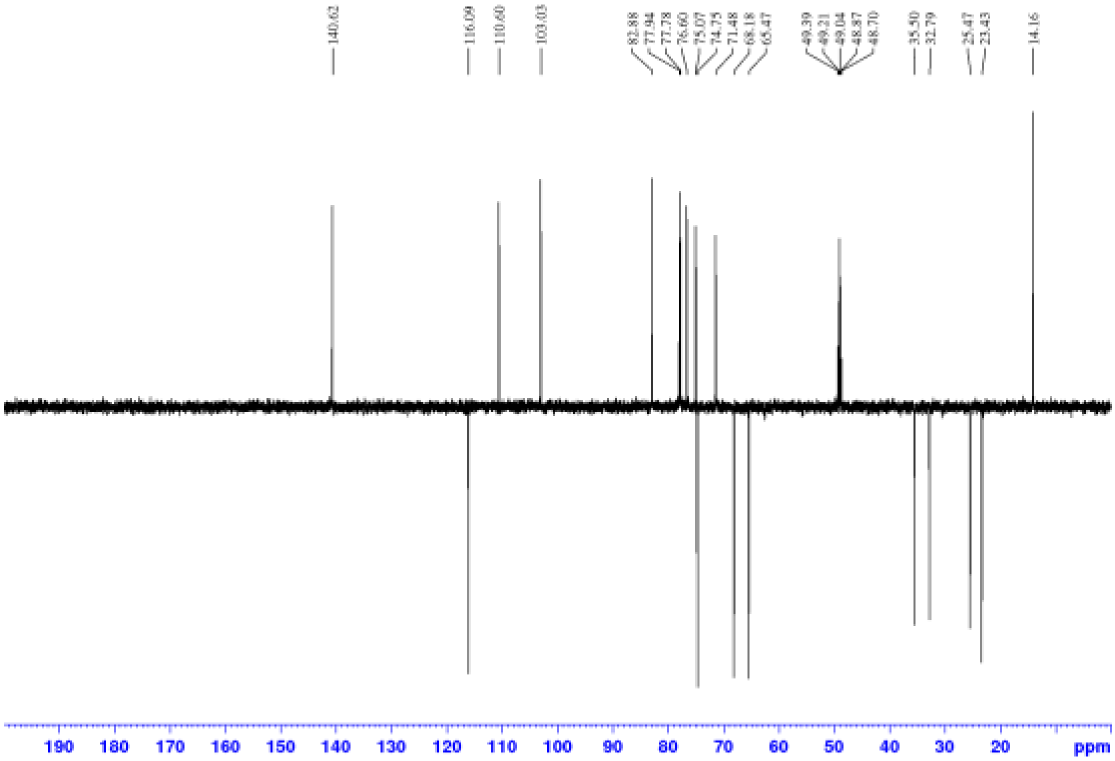

Figure S9: DEPT 135 spectrum of compound **1** (CD_3OD, 125 MHz).

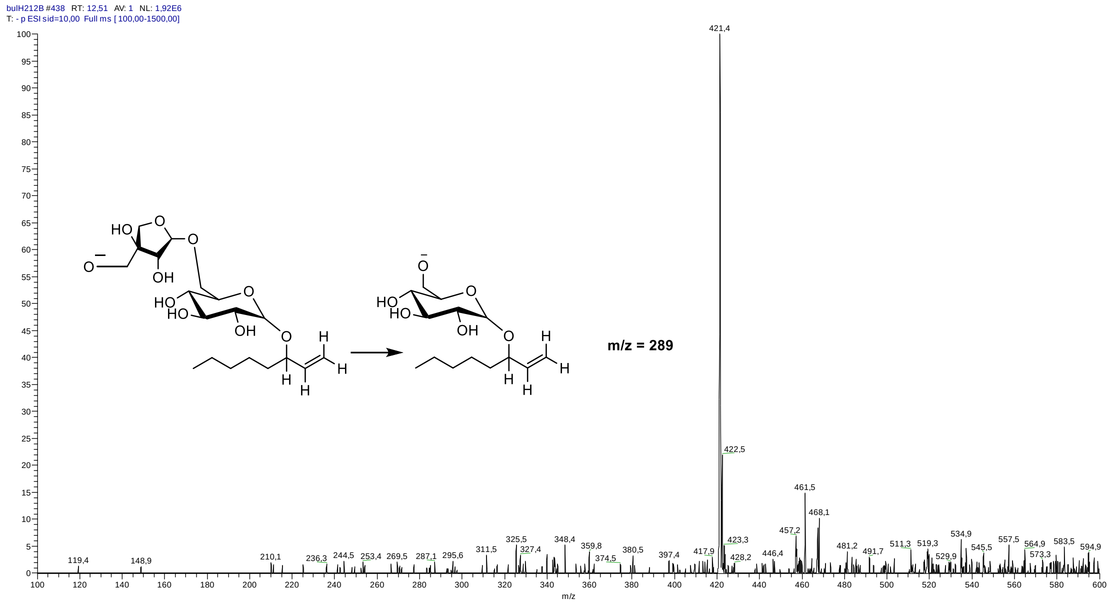

Full mass, (100-1500 *m/z*). [M-H]$^-$: *m/z* 421.4 (100), MS^2 (421.4→289.3 (100), 161.1 (29), 130.9 (8)), MS^3 (289.3→161.3 (100)), MS^4 (161.3→142.6 (100), 73.3 (84)). Mass range: 100-600 *m/z*.

Figure S10: ESI-MS spectrum of compound **1**.

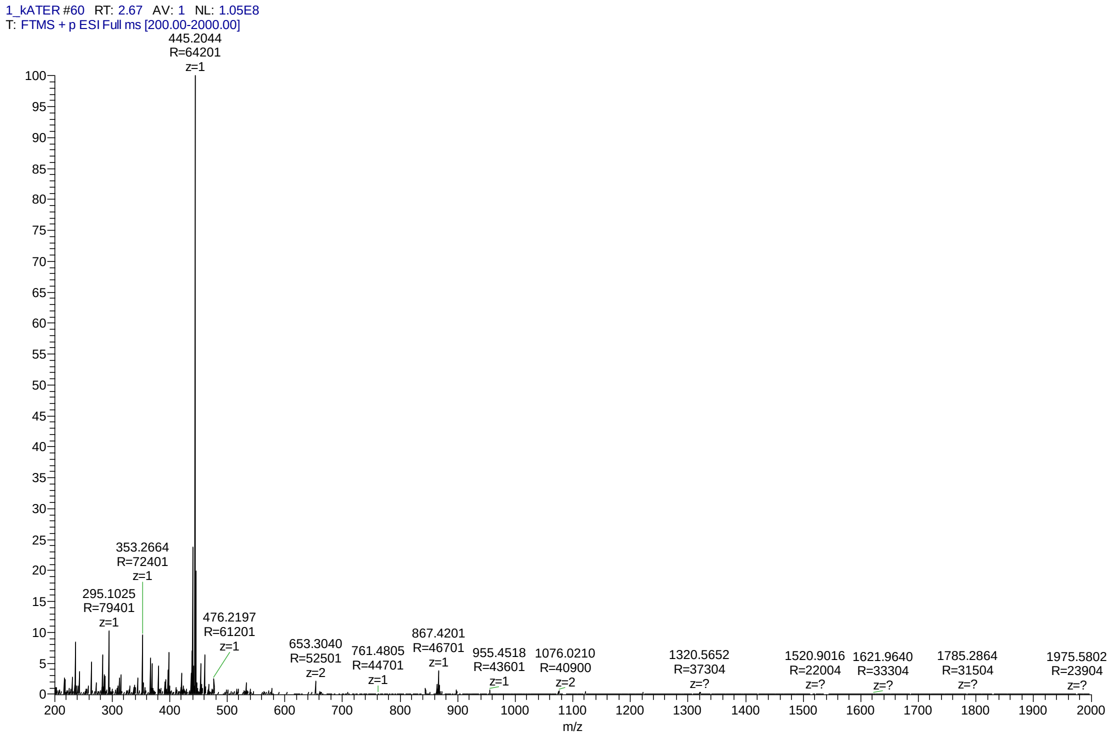

Full mass, (200-2000 *m/z*). [M+Na]$^+$: *m/z* 445.2044 (100)

Figure S11: HRMS spectrum of compound **1**.

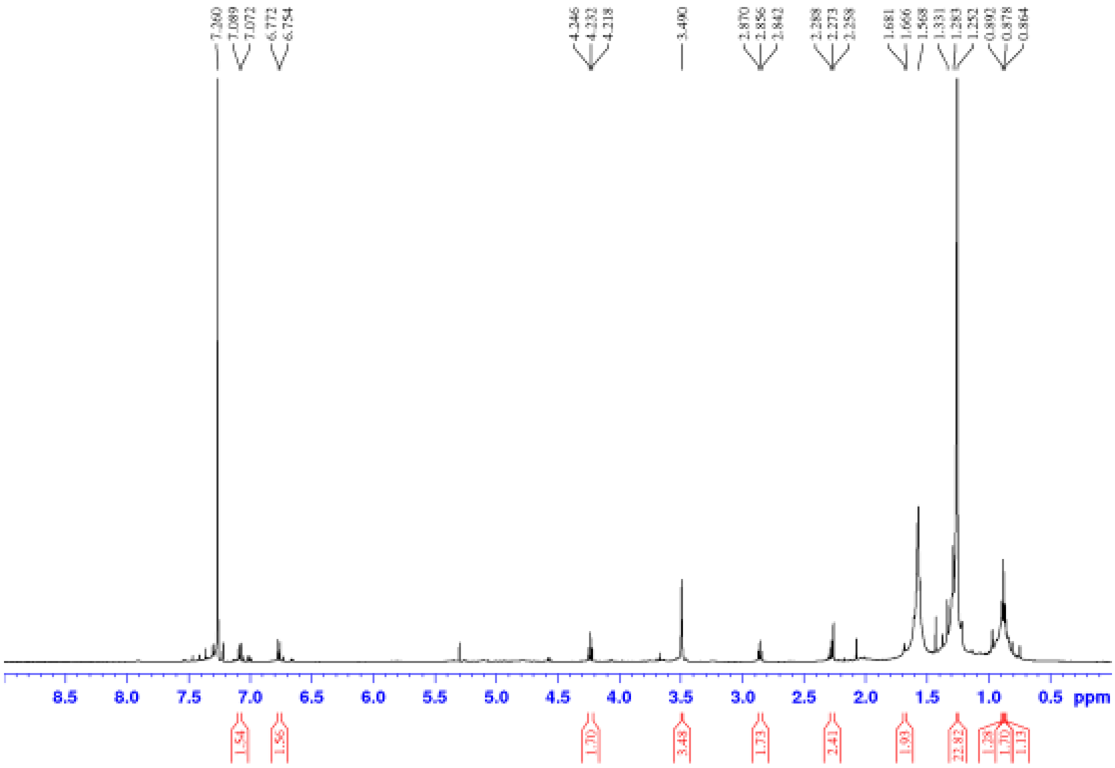

Figure S12: ^{1}H-NMR spectrum of compound **2** ($CDCl_3$, 500 MHz).

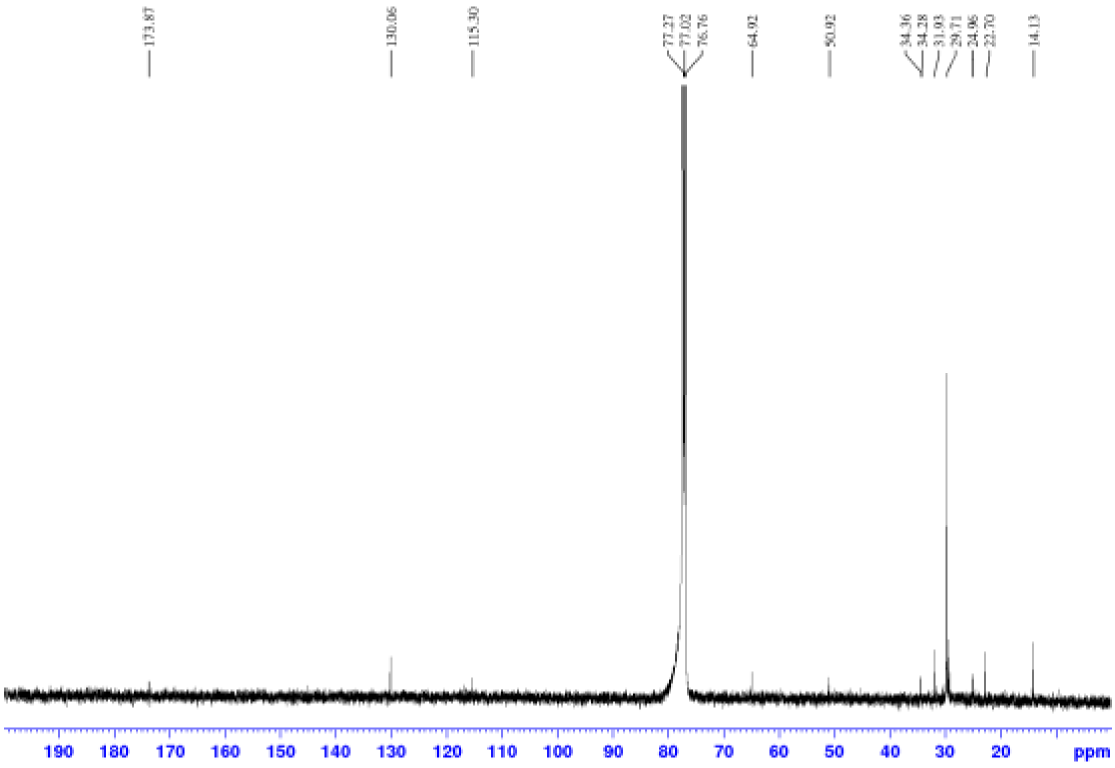

Figure S13: ^{13}C-NMR spectrum of compound **2** ($CDCl_3$, 125 MHz).

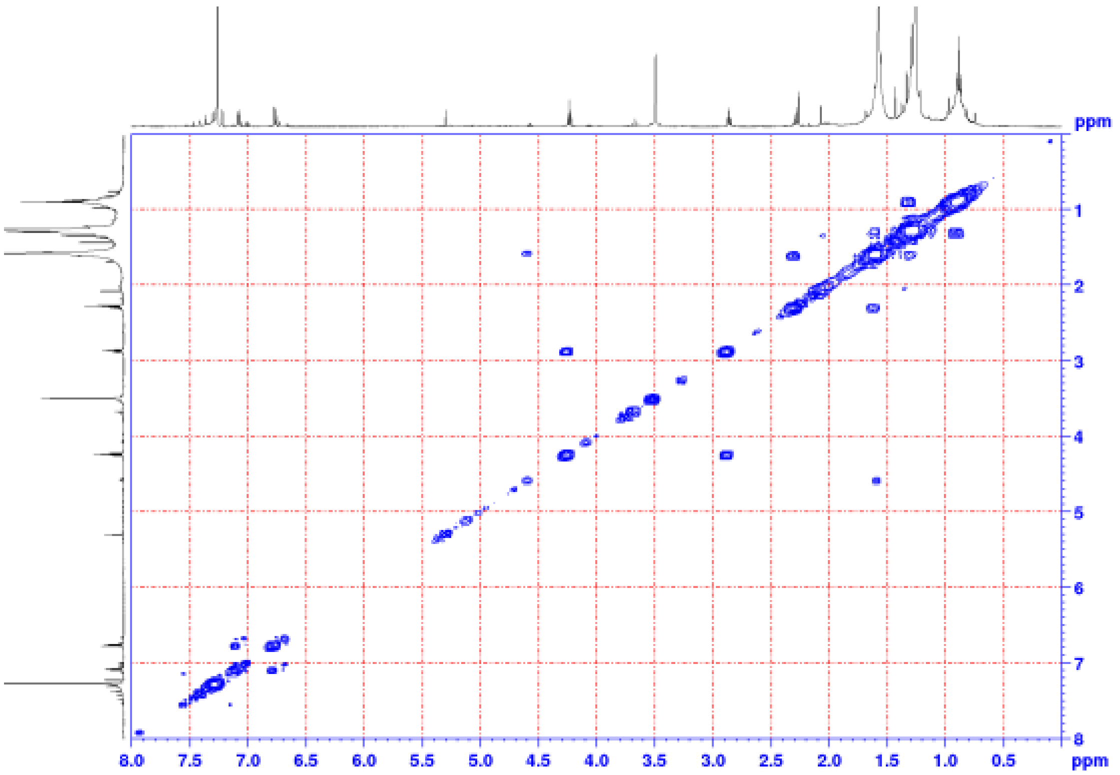

Figure S14: COSY spectrum of compound **2** ($CDCl_3$, 500 MHz).

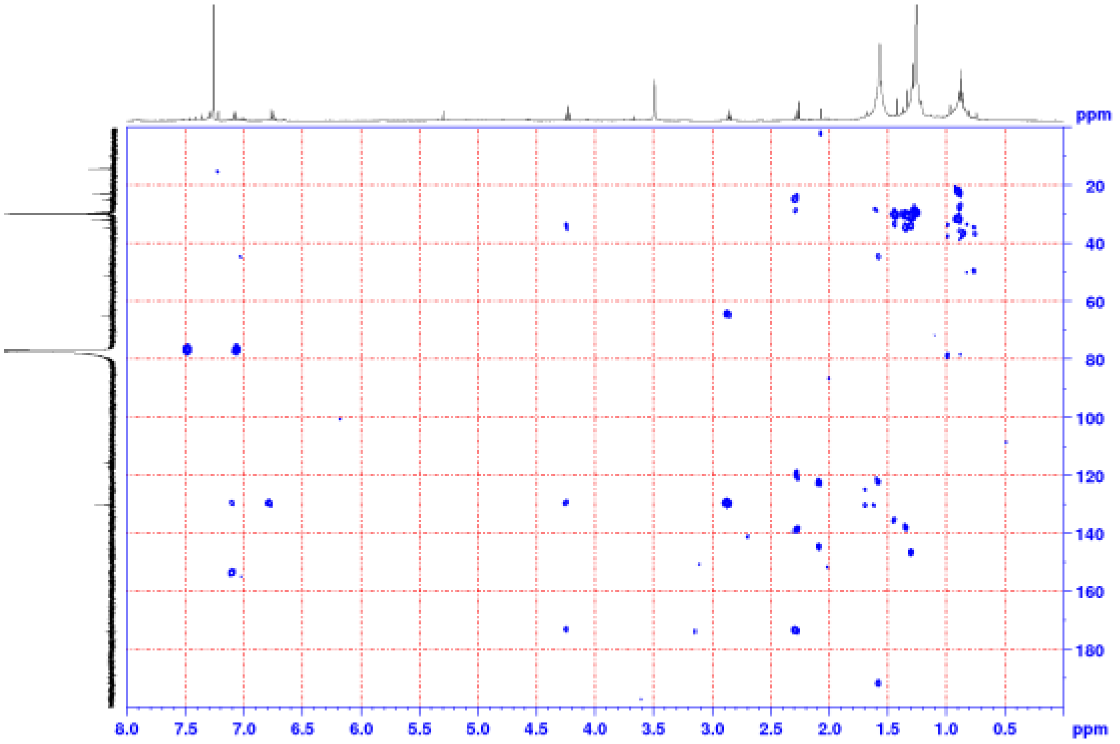

Figure S15: HMBC spectrum of compound **2** ($CDCl_3$, 500 MHz).

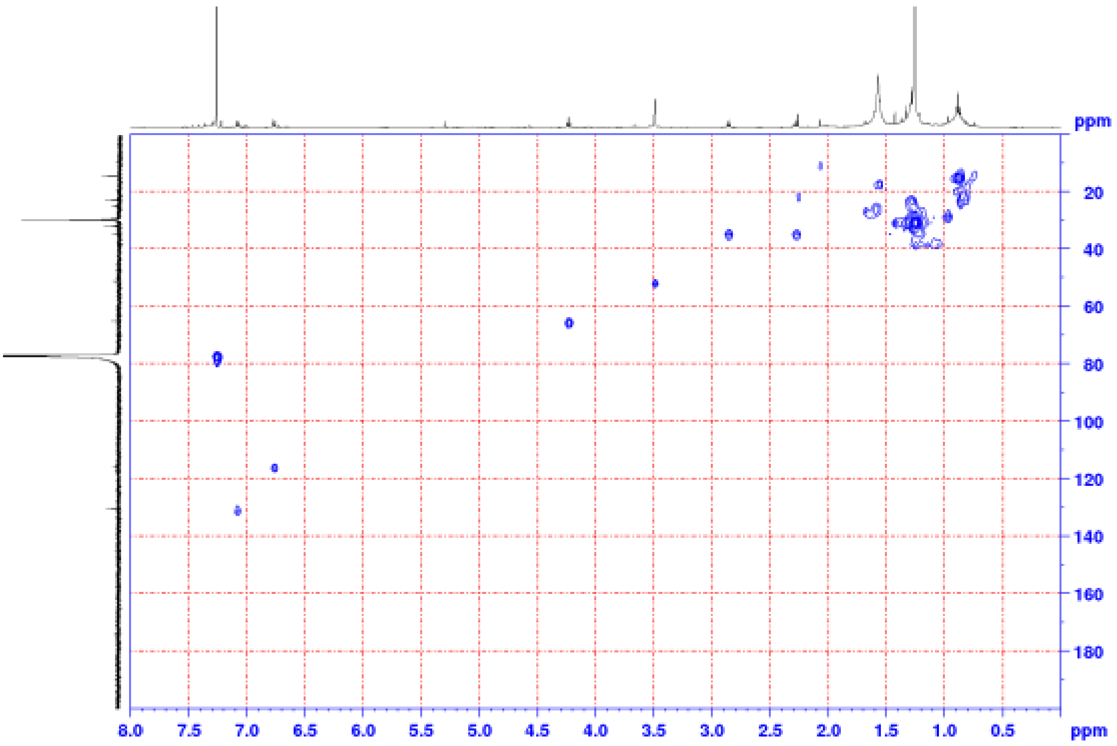

Figure S16: HSQC spectrum of compound **2** ($CDCl_3$, 500 MHz).

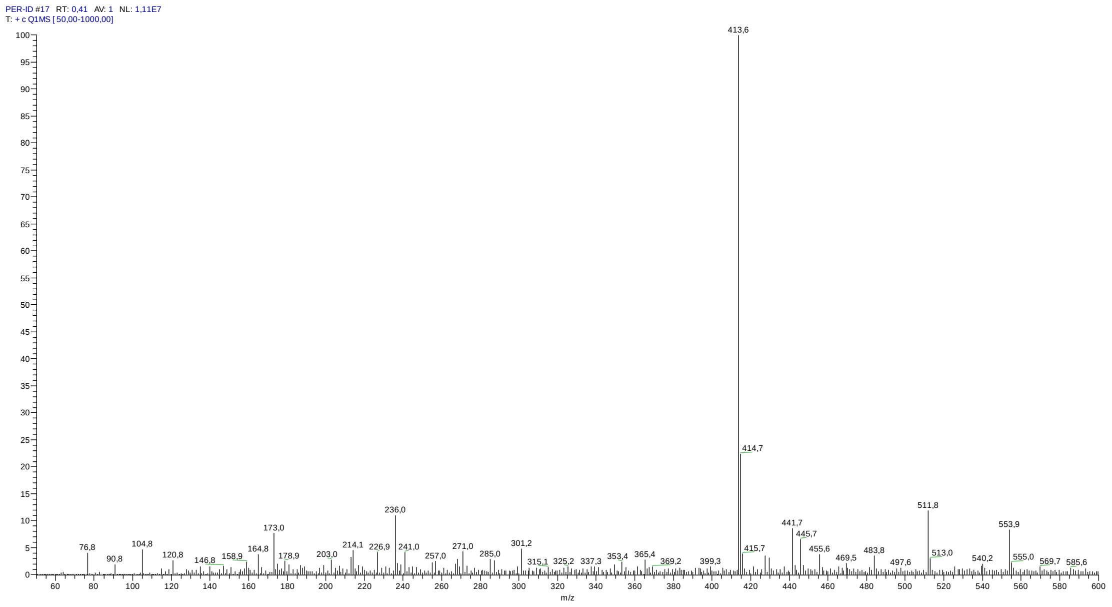

Full mass, (50-1000 *m/z*). $[M+Na]^+$: *m/z* 413.6 (100), MS^2 (413.6→189.0 (100), 165.1 (90), 241.2 (38), 170 (35), 301.1 (30)). Mass range: 80-600 *m/z*.

Figure S17: ESI-MS spectrum of compound **2**.

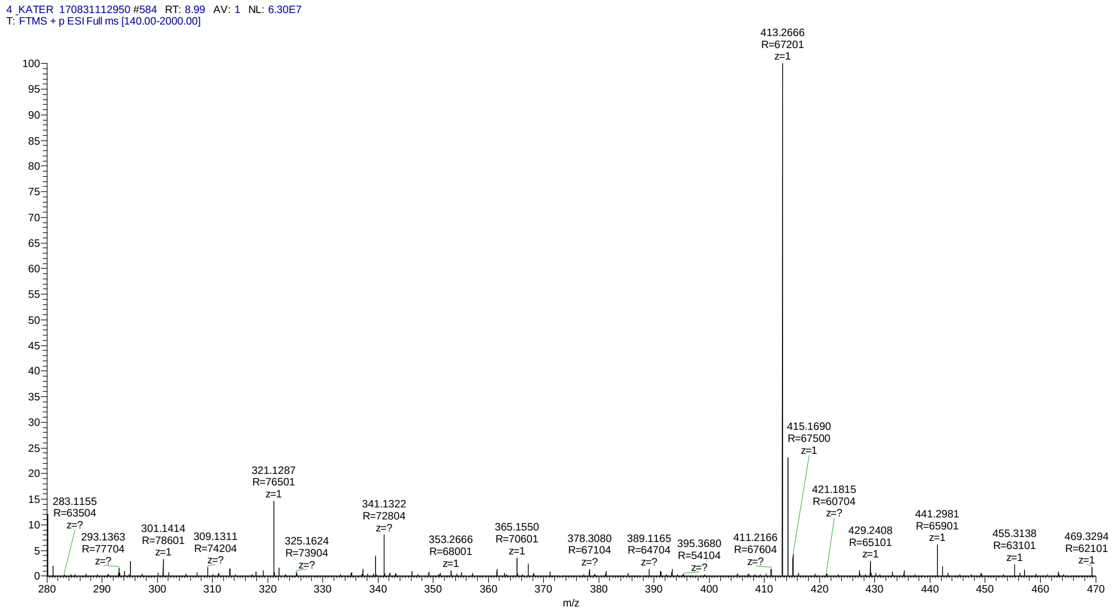

Full mass, (140-2000 *m/z*). $[M+Na]^+$: *m/z* 413.2666 (100). Mass range: 280-470 *m/z*.

Figure S18: HRMS spectrum of compound **2**.

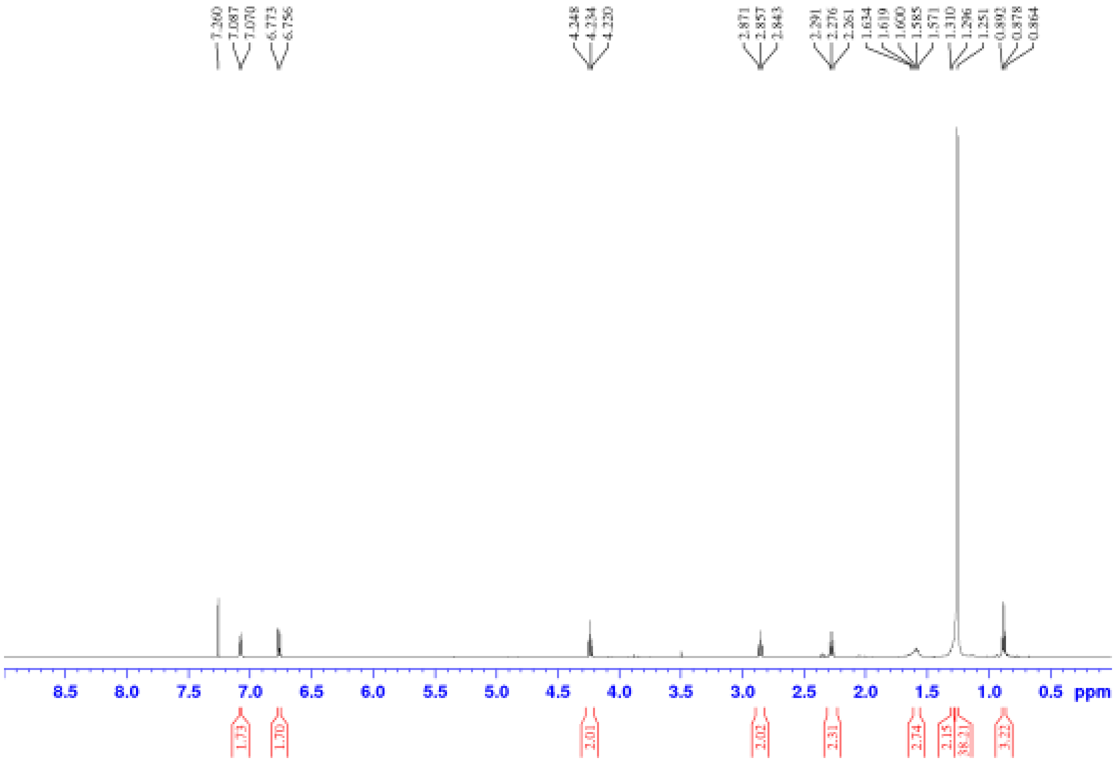

Figure S19: ^{1}H-NMR spectrum of compound **3** ($CDCl_3$, 500 MHz).

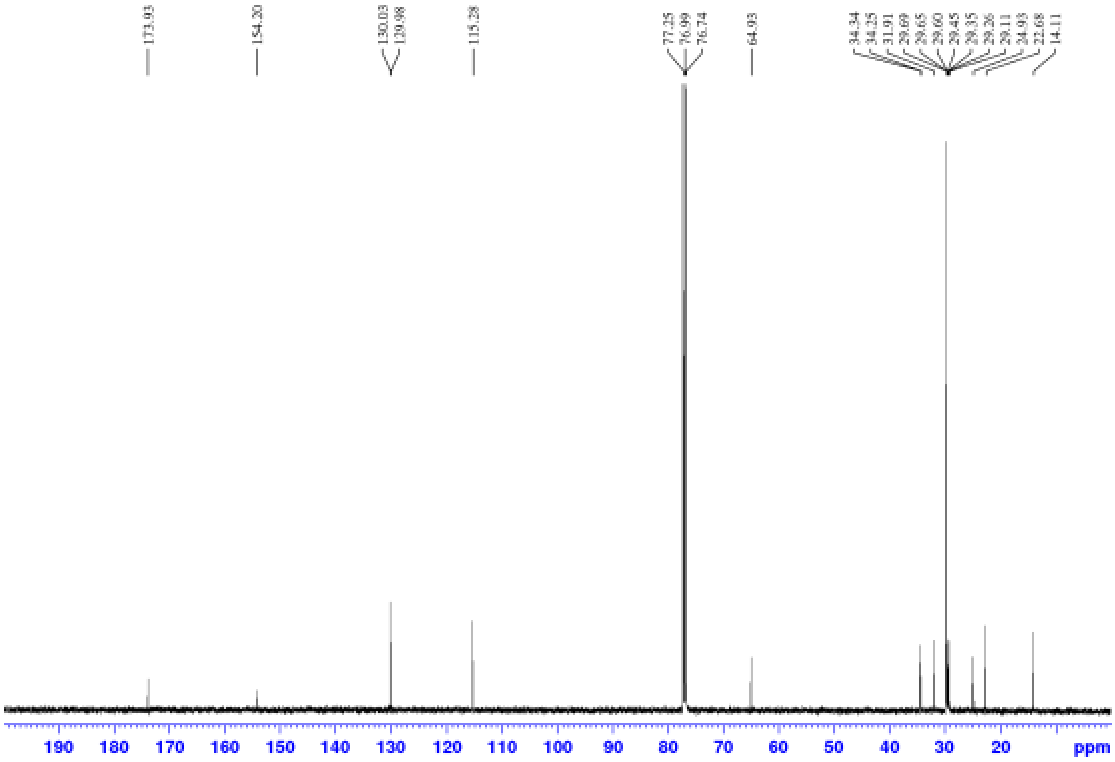

Figure S20: ^{13}C-NMR spectrum of compound **3** ($CDCl_3$, 125 MHz).

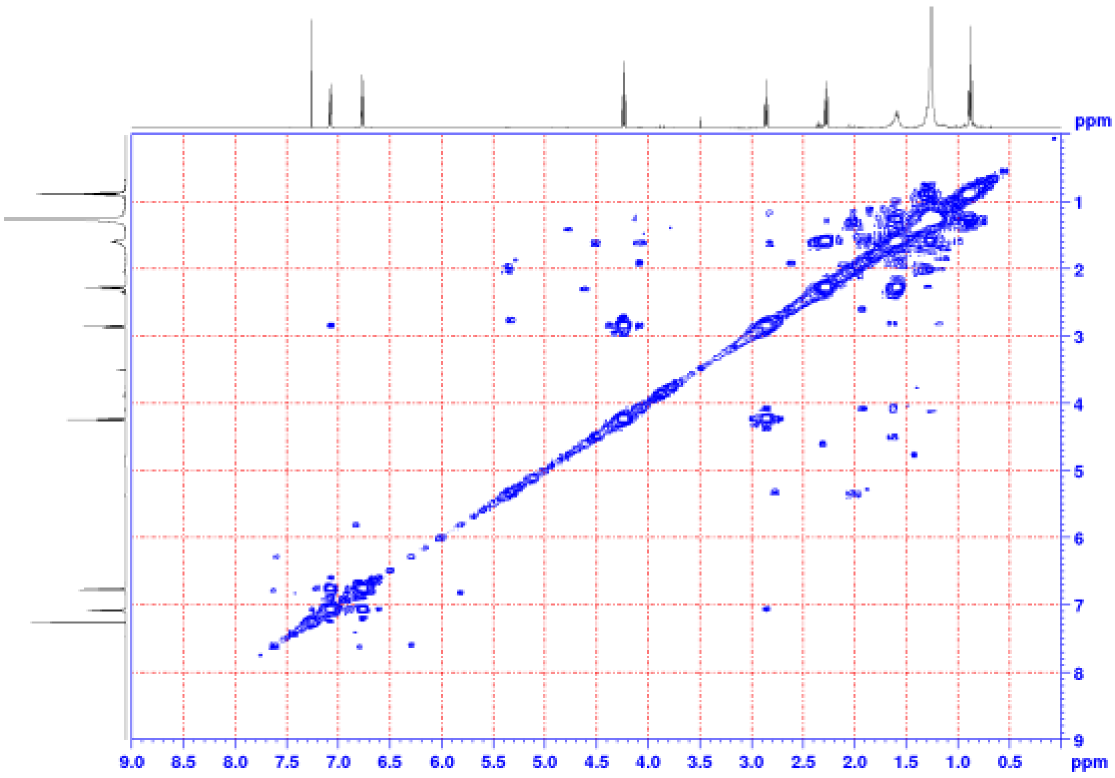

Figure S21: COSY spectrum of compound **3** ($CDCl_3$, 500 MHz).

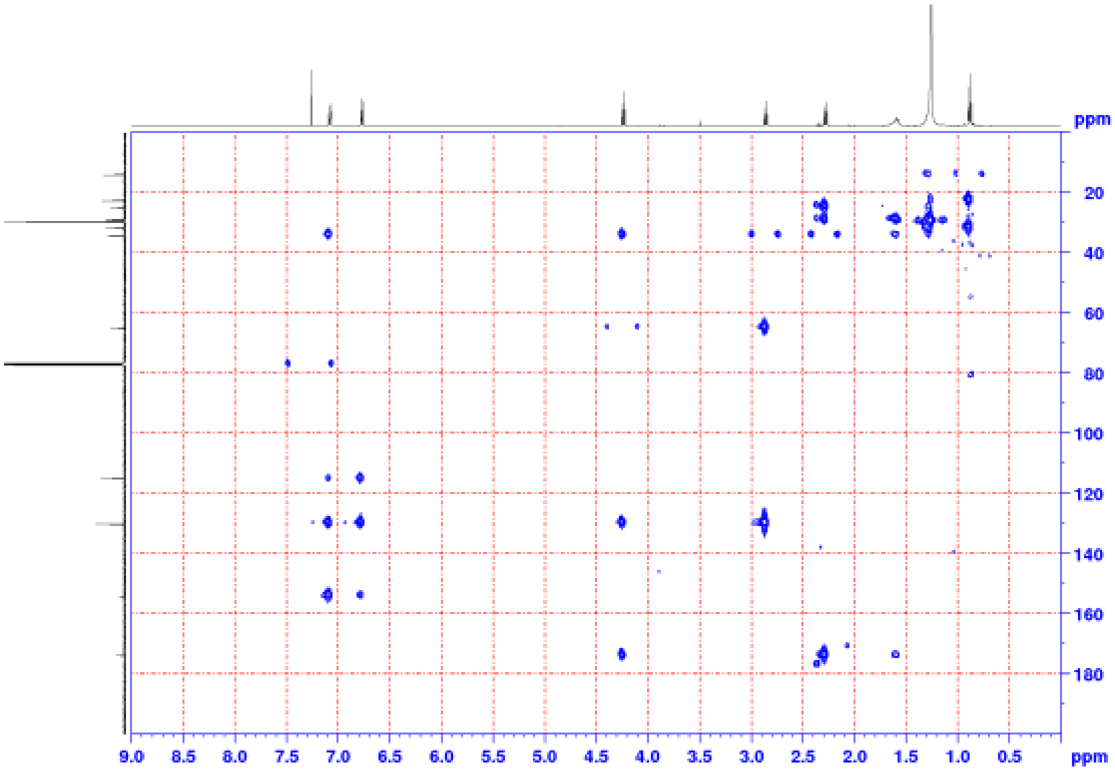

Figure S22: HMBC spectrum of compound **3** ($CDCl_3$, 500 MHz).

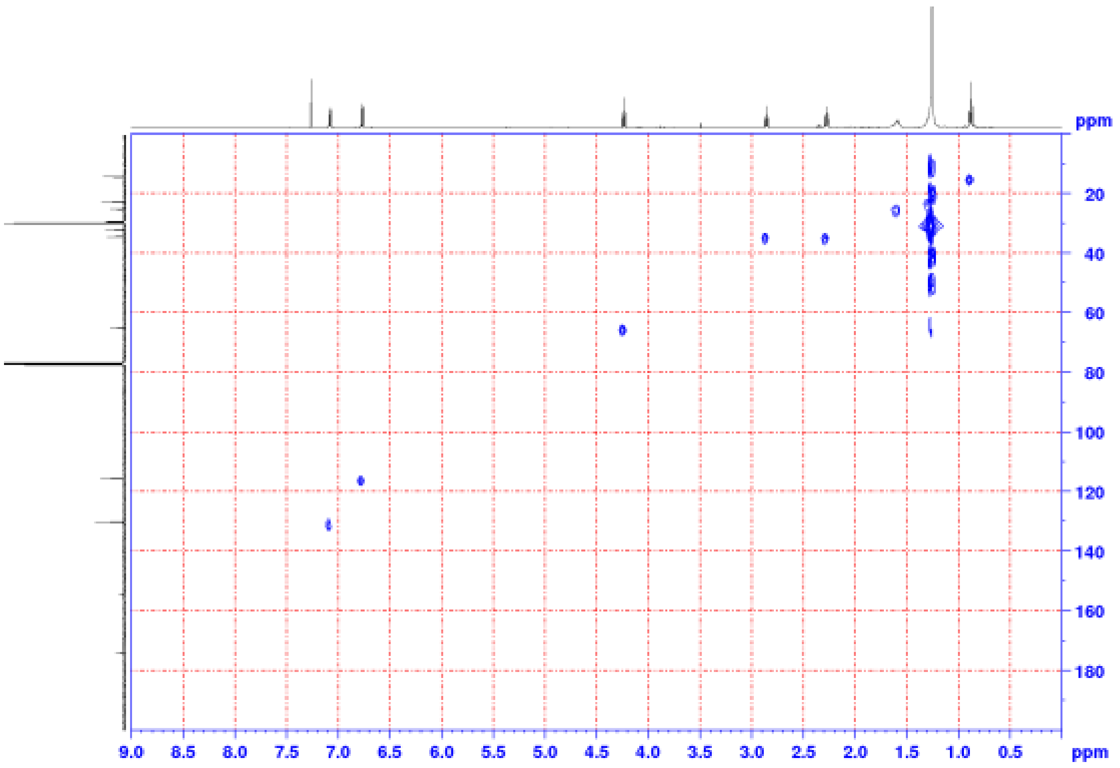

Figure S23: HSQC spectrum of compound **3** ($CDCl_3$, 500 MHz).

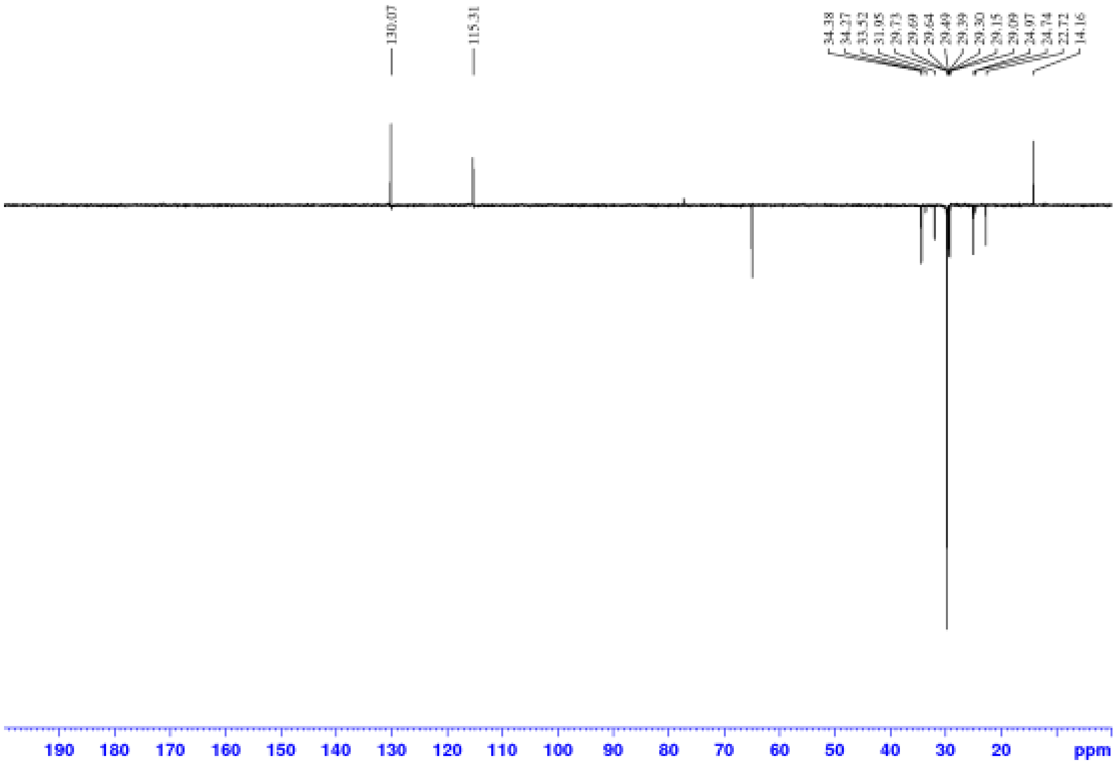

Figure S24: DEPT 135 spectrum of compound **3** ($CDCl_3$, 125 MHz).

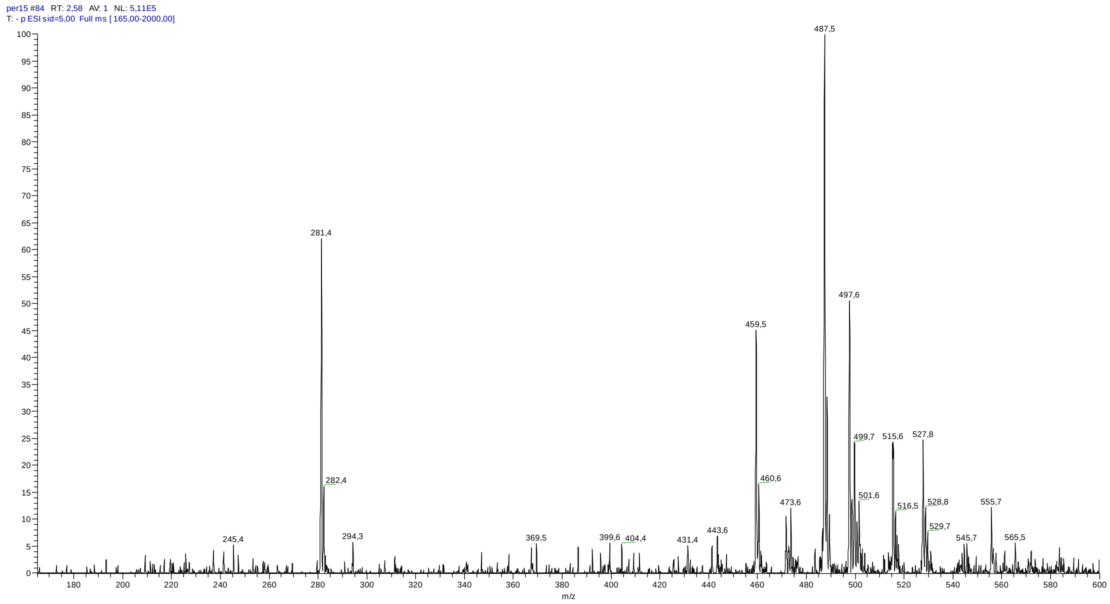

Full mass, (165-2000 *m/z*). [M-H]$^-$: *m/z* 487.5 (100), 281.4 (65), MS^2 (487.5→485.8 (100), 367.6 (60), 281.4 (50)). Mass range: 165-600 *m/z*.

Figure S25: ESI-MS spectrum of compound **3**.

Table S2: NMR spectroscopic data of metabolite **4** (brassicasterol), ($CDCl_3$, 500 MHz).

#	^{13}C	HSQC	DEPT-135	δ ^1H	Multiplicity, (*J* Hz)	COSY	HMBC
1	37.2	H-1a	CH_2	1.86	m, 1H	H-1b	H-19
		H-1b		1.09	m, 1H	H-1a	
2	31.9	H-2a	CH_2	1.84	m, 1H	H-1b, H-2b	H-4a
		H-2b		1.52	m, 1H	H-2a	
3	71.8	H-3	CH	3.51	m, 1H	H-2b, H-4a, H-4b	H-2a, H-2b, H-4a
4	42.3	H-4a	CH_2	2.28	dd, J_1 = 5.3, J_2 = 2.2 Hz, 1H	H-3	H-2a, H-6
		H-4b		2.24	dd, J_1 = 5.0, J_2 = 2.1 Hz, 1H	H-3	
5	140.7	-	C	-	-	-	H-2a, H-2b, H-4a, H-4b, H-19
6	121.7	H-6	CH	5.35	br.d, *J* = 5.3 Hz, 1H	H-4b, H-7a, H-7b	H-4a, H-4b, H-7a, H-7b
7	31.9	H-7a	CH_2	1.97	m, 1H	H-6, H-7b	H-6, H-8
		H-7b		1.53	m, 1H	H-7a	
8	31.6	H-8	CH	1.48	m, 1H	H-7a	H-6, H-7
9	50.1	H-9	CH	0.93	m, 1H	H-8	H-19
10	36.4	-	C	-	-	-	H-4a, H-6, H-9, H-19
11	21.2	H-11	CH_2	1.50	m, 2H	H-9, H-12	H-9
12	40.6	H-12a	CH_2	2.00	m, 1H	H-11, H-12b	

#	^{13}C	HSQC	DEPT-135	δ ^{1}H	Multiplicity, (*J* Hz)	COSY	HMBC
		H-12b		1.18	m, 1H	H-12a	H-18, H-19
13	42.2	-	C	-	-	-	H-18
14	56.7	H-14	CH	1.00	m, 1H	H-8, H-15	H-12, H-18
15	24.3	H-15a	CH_2	1.56	m, 1H	H-14, H-16a	-
		H-15b		1.04	m, 1H	H-16a	
16	28.2	H-16a	CH_2	1.83	m, 1H	H-15a, H-16b, H-17	-
		H-16b		1.25	m, 1H	H-16a	
17	56.0	H-17	CH	1.10	m, 1H	H-16a	H-15b, H-18, H-21
18	11.9	H-18	CH_3	0.67	s, 3H	-	H-17
19	21.1	H-19	CH_3	1.01	s, 3H	-	-
20	39.8	H-20	CH	2.03	m, 1H	-	H-17, H-18
21	18.7	H-21	CH_3	0.91	d, J = 6.5, 3H	-	H-17
22	129.3	H-22	CH	5.01	dd, J_1 = 15.7, J_2 = 8.6 Hz, 1H	H-23	H-23
23	138.3	H-23	CH	5.15	dd, J_1= 15.7, J_2 = 8.6 Hz, 1H	H-22, H-20	H-22
24	39.8	H-24	CH	1.99	ddq, J_1 = 7.8, J_2 = 5.1, J_3 = 2.2 Hz, 1H	H-23	H-28
25	33.9	H-25	CH	1.31	m, 1H	-	H-26, H-27
26	21.1	H-26	CH_3	0.82	d, J = 7.0 Hz, 3H	-	H-27

#	^{13}C	HSQC	DEPT-135	δ ^1H	Multiplicity, (*J* Hz)	COSY	HMBC
27	18.8	H-27	CH_3	0.79	d, J = 7.0 Hz, 3H	-	H-25, H-26
28	11.8	H-28	CH_3	0.68	d, J = 8.9 Hz, 3H	-	H-25

Table S3: NMR spectroscopic data of metabolite **5** (stigmasterol), ($CDCl_3$, 500 MHz).

#	^{13}C	HSQC	DEPT-135	δ ^1H	Multiplicity, (*J*, Hz)	COSY	NOESY	HMBC
1	37.2	H-1a	CH_2	1.07	m, 1H	H-1b, H-2a	H-1b, H-2a	H-19
		H-1b		1.83	m, 1H	H-1a	H-1a	
2	31.6	H-2a	CH_2	1.84	t, J = 4.1 Hz, 1H	H-1a, H-3	H-1a	H-3, H-4a, H-4b
		H-2b		1.97	m, 1H	-	-	
3	71.8	H-3	CH	3.52	tdd, J_1 = 11.1, J_2 = 5.2, J_3 = 4.6 Hz, 1H	H2a, H-4a	H-2a, H-4a, H-4b	H-4a, H-4b, H-1b
4	42.3	H-4a	CH_2	2.24	dd, J_1 = 13.3, J_2 = 2.3 Hz, 1H	H-3	-	H-6
		H-4b		2.28	dd, J_1 = 13.3, J_2 = 2.3 Hz, 1H	H-3	H-6	
5	140.7	-	C	-	-	-	-	H-4a, H-4b, H-7a, H-8, H-19
6	121.7	H-6	CH	5.34	m, 1H	H4a, H7b	H-4b, H-7b, H-8	H-4a, H-4b, H-8
7	31.6	H-7a	CH_2	1.52	m, 1H	H-8	H-8	H-4b
		H-7b		1.97	m, 1H	H-8	H-14, H-12b, H-8	
8	31.8	H-8	CH	1.52-1.50	m, 1H	H-7a, H-7b, H-9	H-7a, H-7b	H-9

#	^{13}C	HSQC	DEPT-135	δ ^1H	Multiplicity, (*J*, Hz)	COSY	NOESY	HMBC
9	50.1	H-9	CH	0.94	m 1H	H-8	H-8	H-12a, H-14
10	36.5	-	C	-	-	-	-	H-2, H-4, H-6, H-19
11	21.1	H-11	CH_2	1.49-1.51	m, 2H	H-9, H-12a	H-12a	H-9
12	39.7	H-12a	CH_2	1.99-2.01	m, 1H	H-11, H-12b	H-12b, H-18	H-14, H-18
		H-12b		1.17-1.19	m, 1H	H-11, H-12a	H-12a	
13	42.1	-	C	-	-	-	-	H-18
14	56.8	H-14	CH	1.01	br. d, J = 2.0 Hz, 1H	H-8, H-15a	H-15	H-12a, H-18
15	24.3	H-15a	CH_2	1.55	m, 1H	H-14	H-14	H-16b
		H-15b		1.06	m, 1H	-	H-14	
16	28.9	H-16a	CH_2	1.24-1.28	m, 1H	H-16b, H-17	H-16b	H-17
		H-16b		1.69	ddt, J_1 = 13.9, J_2 = 5.7, J_3 = 3.5, 1H	H-16a	H-16a	
17	55.9	H-17	CH	1.13-1.15	m, 1H	H-20	H-20	H-12, H-18, H-16, H-20, H-21
18	12.0	H-18	CH_3	0.69	s, 3H	-	H-11, H-12, H-21	H-12a, H-14
19	19.4	H-19	CH_3	1.00	s, 3H	-	-	H-20
20	40.5	H-20	CH	1.13-1.15	m, 1H	H-21	H-17	H-21, H-22
21	21.1	H-21	CH_3	1.02	d, J = 6.7 Hz, 3H	-	H-24	H-22
22	138.3	H-22	CH	5.14	dd, J_1 = 15.5, J_2 = 8.7 Hz, 1H	H-23	H-21,H-24, H-28b, H-29	H-20, H-21, H-23
23	129.2	H-23	CH	5.01	dd, J_1 = 15.5, J_2 = 8.7 Hz, 1H	H-22, H-24	H-21, H-24, H-28b, H-29	H-22, H-24, H-28a, H-28b H-26

#	^{13}C	HSQC	DEPT-135	δ ^1H	Multiplicity, (*J*, Hz)	COSY	NOESY	HMBC
								H-27
24	51.2	H-24	CH	1.52	m, 1H	H-23, H-25, H-28	H-29, H-21 H-25	H-22, H-23, H-26, H-27
25	31.9	H-25	CH	1.44	m, 1H	H-24	-	H-26, H-28a, H-24
26	21.2	H-26	CH_3	0.84	d, *J* = 6.4 Hz, 3H	-	H-24	H-24, H-27
27	19.0	H-27	CH_3	0.79	d, *J* = 6.4 Hz, 3H	-	H-24, H-28, H-29	H-24, H-26
28	25.4	H-28a	CH_2	1.41	m, 1H	H-24, H-28b, H-29	H-28b	H-24, H-29
		H-28b		1.16	m, 1H	H-28a, H-29	H-27	
29	12.2	H-29	CH_3	0.81	t, *J* = 7.4 Hz, 3H	H-28a, H-28b	H-24	H-28b

Table S4: NMR spectroscopic data of metabolite **6** (verbascoside), (CD_3OD, 500 MHz).

#	^{13}C	HSQC	DEPT-135	δ ^1H	Multiplicity, (*J*, Hz)	COSY	NOESY	HMBC
**Phenethyl group**								
1	131.4	-	C	-	-	-	-	H-5, H-7, H-8a, H-8b
2	117.1	H-2	CH	6.69	d, *J* = 2.0 Hz, 1H	H-6	H-7	H-6, H-7
3	146.0		C	-	-	-	-	H-5
4	144.5		C	-	-	-	-	H-2, H-6
5	117.2	H-5	CH	6.67	d, *J* = 8.0 Hz, 1H	H-6	-	-

#	^{13}C	HSQC	DEPT-135	δ ^1H	Multiplicity, (*J*, Hz)	COSY	NOESY	HMBC
6	121.3	H-6	CH	6.56	dd, J_1 = 8.0, J_2 = 2.0 Hz, 1H	H-2, H-5	-	H-2, H-5, H-7
7	36.4	H-7	CH_2	2.79	ddt, J_1 = 3.5, J_2 = 7.3, J_3 = 14.0 Hz, 2H	H-8a, H-8b	H-2, H-6, H-8a, H-8b	H-8, H-2, H-6
8	71.9	H-8a	CH_2	4.04	m, 1H	H-7, H-8b	H-8	H-7, H-1″
		H-8b		3.73	m, 1H	H-7, H-8a	H-7	H-7, H-1″
**Caffeoyl group**								
1′	127.6	-	C	-	-	-	-	H-5′, H-7′, H-8′
2′	114.6	H-2′	CH	7.05	d, *J* = 2.0 Hz, 1H	H-6′	-	H-6′, H-7′
3′	146.7	-	C	-	-	-	-	H-2′, H-5′
4′	149.7	-	C	-	-	-	-	H-2′, H-5′, H-6′
5′	116.5	H-5′	CH	6.78	d, *J* = 8.1 Hz, 1H	H-6′	-	H-6′, H-7′
6′	123.3	H-6′	CH	6.95	dd, J_1 = 8.3, J_2 = 2.0 Hz, 1H	H-2′, H-5′	-	H-2′, H-5′, H-7′
7′	148.0	H-7′	CH	7.59	d, *J* = 16.0 Hz, 1H	H-8′	H-8′	H-2′, H-6′
8′	114.6	H-8′	CH	6.28	d, *J* = 16.0 Hz, 1H	H-7′	H-7′	H-7′
9′	168.4	-	C=O	-	-	-	-	H-7′, H-8′, H-4″
***β*-D-glucose**								
1″	104.0	H-1″	CH	4.38	d, *J* = 7.8 Hz, 1H	H-2″	H-2″	H-2″, H-8b
2″	76.1	H-2″	CH	3.39	dd, J_1 = 9.0, J_2 = 8.0 Hz, 1H	H-1″, H-3″	-	H-3″, H-4″, H-5″

#	^{13}C	HSQC	DEPT-135	δ ^{1}H	Multiplicity, (*J*, Hz)	COSY	NOESY	HMBC
3″	81.7	H-3″	CH	3.81	t, *J* = 9.3 Hz, 1H	H-4″	H-1‴	H-2″, H-4″, H-1‴
4″	70.5	H-4″	CH	4.94	t, *J* = 9.8 Hz, 1H	H-3″, *H-5″	-	H-3″, H-5″, H-1‴
5″	75.8	H-4″	CH	3.54	m, 1H	H-4″, H-6″	-	H-3″, H-4″
6″	62.2	H-6a″	CH_2	3.63	d, *J* = 10.0 Hz, 1H	*H-5″, H-6b″	-	H-4″
		H-6b″		3.51	d, *J* = 10.0 Hz, 1H	*H-5″, H-6a″	-	H-4″
**α-L-Rhamnose**								
1‴	103.0	H-1‴	CH	5.18	d, *J* = 1.5 Hz, 1H	H-2‴	H-3″, H-2‴	H-3″
2‴	72.3	H-2‴	CH	3.92	dd, J_1 = 3.1, J_2 = 1.6 Hz, 1H	H-1‴	H-1‴	H-1‴, *H-4‴
3‴	72.2	H-3‴	CH	3.57	dd, J_1 = 3.5, J_2 = 9.6 Hz, 1H	H-4‴, *H-5‴	H-5‴	H-1‴, *H-4‴
4‴	73.7	H-4‴	CH	*3.28	d, *J* = 9.6 Hz, 1H	*H-5‴	H-6‴	H-2‴, H-5‴
5‴	70.3	H-5‴	CH	*3.52	m, 1H	*H-4‴, *H-6‴	H-6‴	H-1‴, H-6‴
6‴	18.4	H-6‴	CH_3	1.09	d, *J* = 6.2 Hz, 3H	*H-5‴	H-4‴, *H-5‴	H-4‴

* Unclear due to overlapping

Table S5: NMR spectroscopic data of metabolite **7** (martynoside), (CD_3OD, 500 MHz).

#	^{13}C	HSQC	DEPT 135	δ ^{1}H	Multiplicity, (*J*, Hz)	COSY	NOESY	HMBC
**Phenethyl group**								

#	^{13}C	HSQC	DEPT 135	δ ^{1}H	Multiplicity, (J, Hz)	COSY	NOESY	HMBC
1	132.9	-	C	-	-	-	-	H-5, H-7
2	117.1	H-2	CH	6.74	d, J = 2.0 Hz, 1H	-	-	H-7
3	147.5	-	C	-	-	-	-	H-2, H-5, H-6, 4-OCH_3
4	147.6	-	C	-	-	-	-	H-2, H-5, H-6, 4-OCH_3
5	112.9	H-5	CH	7.19	d, J = 6.6 Hz, 1H	H-6	H-6, 4-OCH_3	-
6	121.2	H-6	CH	6.69	dd, J_1 = 8.0, J_2 = 2.1 Hz, 1H	H-5	H-5	H-2, H-5, H-7
7	36.6	H-7	CH_2	2.81	m, 2H	H-8a, H-8b	H-8a, H-8b	H-2
8	72.4	H-8a	CH_2	4.03	m, 1 H	H-7, H-8b	H-7, H-8b	H-1″, H-7
		H-8b		3.75	m, 1 H	H-7, H-8a	H-7, H-8a	
**Feruloyl group**								
1′	127.7	-	C	-	-	-	-	H-5′, H-8′
2′	111.8	H-2′	CH	7.20	d, J = 2.0 Hz, 1H	-	3′-OCH_3	H-6′, H- 7′
3′	149.4	-	C	-	-	-	-	H-2′, H- 5′, 3′-OCH_3
4′	150.8	-	C	-	-	-	-	H-2′, H- 5′, H-6′
5′	116.5	H-5′	CH	6.84	d, J = 7.2 Hz, 1H	H-6′	H-6′	-
6′	124.4	H-6′	CH	7.09	dd, J_1 = 8.3, J_2 = 2.0 Hz, 1H	H-5′	H-5′	H-2′
7′	147.4	H-7′	CH	7.66	d, J = 16.0 Hz, 1H	H-8′	H-8′	H-2′, H-6′

#	^{13}C	HSQC	DEPT 135	δ ^{1}H	Multiplicity, (*J*, Hz)	COSY	NOESY	HMBC
8′	115.2	H-8′	CH	6.37	d, J = 16.0 Hz, 1H	H-7′	H-7′	-
9′	168.2	-	C=O	-	-	-	-	H-7′, H-8′, H-4″
				***β*-D-Glucose**				
1″	104.3	H-1″	CH	4.37	d, J = 8.0 Hz, 1H	H-2″	-	H-2″
2″	76.2	H-2″	CH	3.38	dd, J_1 = 9.2, J_2 = 7.8 Hz, 1H	H-1″	-	H-3″
3″	80.6	H-3″	CH	3.80*	m, 1H*	H-4″*	-	H-2′, H-1‴
4″	70.4	H-4″	CH	4.92	*	H-3″	-	H-3′, H-1‴
5″	75.1	H-5″	CH	3.53	m, 1H*	-	-	H-3′, H-4″
6″	62.4	H-6a″	CH_2	3.64	m, 1H*	H-6b″	*	-
		H-6b″		3.53	m, 1H*	H-6a″	*	-
				***α*-L-Rhamose**				
1‴	103.0	H-1‴	CH	5.19	d, J = 2.0 Hz, 1H	-	*H-3″, H-4‴	*H-3″
2‴	72.3	H-2‴	CH	3.91	dd, J_1 = 5.5, J_2 = 2.5 Hz, 1H	H-1‴	H-1‴	H-1‴
3‴	72.0	H-3‴	CH	3.56	m, 1H*	-	-	-
4‴	73.7	H-4‴	CH	3.29	m, 1H*	-	-	H-6‴
5‴	70.9	H-5‴	CH	3.57	m, 1H*	-	-	H-6‴
6‴	18.4	H-6‴	CH_3	1.09	d, J = 6.2 Hz, 3H	H-5‴	H-5‴	H-4‴

#	^{13}C	HSQC	DEPT 135	δ ^1H	Multiplicity, (*J*, Hz)	COSY	NOESY	HMBC
4-OCH_3	56.5	4-OCH_3	CH_3	3.81	s, 3H	-	-	-
3′-OCH_3	56.5	3′-OCH_3	CH_3	3.89	s, 3H	-	-	-

* Unclear due to overlapping

Table S6: NMR spectroscopic data of metabolite **8** (forsythoside B), (CD_3OD, 500 MHz).

#	^{13}C	HSQC	DEPT-135	δ ^1H	Multiplicity, (*J*, Hz)	COSY	NOESY	HMBC
**Phenethyl group**								
1	131.4	-	C	-	-	-	-	H-5, H-7, H-8a, H-8b
2	117.1	H-2	CH	6.70	d, *J* = 2.0 Hz, 1H	H-6	H-7, H-8a, H-8b	H-6, H-7
3	146.1	-	C	-	-	-	-	H-2, H-5, H-6
4	144.7	-	C	-	-	-	-	H-2, H-6
5	116.5	H-5	CH	6.68	d, *J* = 8.0 Hz, 1H	H-6	-	H-6
6	121.3	H-6	CH	6.57	dd, J_1 = 8.0, J_2 = 2.0 Hz, 1H	H-2, H-5	H-7, H-8a, H-8b	H-2, H-5
7	36.6	H-7	CH_2	2.80	m, 2H	H-8a, H-8b	H-2, H-6, H-8a, H-8b	H-6, H-2, H-8a, H-8b
8	72.1	H-8a	CH_2	4.00	m, 1H	H-7, H-8b	H-2, H-6, H-7, H-8b	H-1″, H-7
		H-8b		3.72	m, 1H	H-7, H-8a	H-2, H-6, H-7, H-8a	H-1″, H-7
**Caffeoyl group**								
1′	127.6	-	C	-	-	-	-	H-5′, H-7′, H-8′

#	^{13}C	HSQC	DEPT-135	δ ^{1}H	Multiplicity, (J, Hz)	COSY	NOESY	HMBC
2′	114.7	H-2′	CH	7.06	d, J = 2.0 Hz, 1H	H-6′	-	-
3′	146.8	-	C	-	-	-	-	H-2′, H-5′
4′	149.8	-	C	-	-	-	-	H-2′, H-5′, H-6′
5′	117.2	H-5′	CH	6.78	d, J = 8.2 Hz, 1H	H-6′-	-	H-6′
6′	123.3	H-6′	CH	6.96	dd, J_1 = 8.2, J_2 = 2.0 Hz, 1H	H-5′, H-2′	-	H-2′, H-7′
7′	148.1	H-7′	CH	7.60	d, J = 15.7 Hz, 1H	H-8′, H-6′	H-8′	H-2′, H-6′
8′	115.2	H-8′	CH	6.28	d, J = 15.7 Hz, 1H	H-7′-	H-7′	H-6′, H-7′
9′	168.2	-	C=O	-	-	-	-	H-7′, H-8′, H-4″
***β*-D-Glucose**								
1″	104.3	H-1″	CH	4.37	d, J = 7.9 Hz, 1H	H-2″,	H-2″	H-8a, H-2″, H-5″
2″	76.2	H-2″	CH	3.38	dd, J_1 = 8.9, J_2 = 8.2 Hz, 1H	H-1″, H-3″	H-1″	H-3″
3″	81.7	H-3″	CH	3.80	t, J = 9.3 Hz, 1H	H-2″, H-4″	H-1‴	H-1‴, H-2″, H-4″
4″	70.5	H-4″	CH	4.94	t, J = 9.4 Hz, 1H	H-3″	-	H-1‴, H-3″
5″	75.1	H-5″	CH	3.73	m, 1H overlap	H-4″, H-6b″		H-4″
6″	68.5	H-6a″	CH$_2$	3.74	m, 1H	H-5″, H-6b″		H-4″, H-1⁗
		H-6b″		3.48	dd, J_1 = 11.6 Hz, J_2 = 6.42 Hz, 1H	H-5″, H-6a″	H-1⁗	H-4″, H-1⁗
***α*-L-Rhamnose**								

#	^{13}C	HSQC	DEPT-135	δ ^{1}H	Multiplicity, (J, Hz)	COSY	NOESY	HMBC
1‴	103.1	H-1‴	CH	5.18	d, J = 1.5 Hz, 1H	H-2‴	H-3″, H-4″	H-3″
2‴	72.4	H-2‴	CH	3.91	m, 1H	H-1‴, H-3‴	H-1‴, H-3″	-
3‴	72.3	H-3‴	CH	3.55	dd, J_1 = 9.35, J_2 = 3.4 Hz, 1H	H-2‴, H-4‴		-
4‴	73.8	H-4‴	CH	3.29	*d, J = 9.4 Hz, 1H	H-3‴, H-5‴	H-6‴	H-5‴, H-6‴
5‴	70.9	H-5‴	CH	3.56	*m, 1H	H-4‴, H-6‴	H-6‴	H-1‴, H-6‴, H-3‴
6‴	18.5	H-6‴	CH_3	1.08	d, J = 6.3 Hz, 3H	H-5‴	H-3‴, H-5‴	H-4‴
				**β-D-Apiose**				
1⁗	111.1	H-1⁗	CH	4.90	d, J = 2.2 Hz, 1H	H-4b⁗, H-2⁗	H-6b″, H-4b⁗, H-2⁗	H-2⁗, H-4b⁗,H-6a″, H-6b″
2⁗	78.1	H-2⁗	CH	3.86	d, J = 2.2 Hz, 1H	H-1⁗	H-1⁗	H-5⁗, H-4b⁗
3⁗	80.6	-	C	-	-	-	-	H-4b⁗
4⁗	75.1	H-4a⁗	CH_2	3.91	br d, J = 9.6 Hz, 1H	H-4b⁗, H-5⁗	H-4b⁗	H-5⁗
		H-4b⁗		3.73	d, J = 3.4 Hz, 1H	H-4a⁗, H-5⁗	H-4a⁗	H-5⁗
5⁗	65.7	H-5⁗	CH_2	3.54	br.s, 2H	H-4a⁗, H-4b⁗	H-2⁗	H-4a⁗, H-2⁗

* Unclear due to overlapping

Table S7: NMR spectroscopic data of metabolite **9** (allysonoside), (CD_3OD, 500 MHz).

#	†^{13}C	HSQC	δ ^{1}H	Multiplicity , (J Hz)	COSY	HMBC
**Phenethyl group**						
1	131.4	-	-	-	-	H-5, H-7, H-8a, H-8b
2	117.1	H-2	6.70	d, J = 2.1 Hz, 1H	H-6	H-6, H-7
3	146.8	-	-	-	-	H-2, H-6, H-5
4	144.7	-	-	-	-	H-2, H-6
5	116.9	H-5	6.68	d, J = 8.0 Hz, 1H	H-6	H-6
6	116.7	H-6	6.57	dd, J_1 = 8.0, J_2 = 2.0 Hz, 1H	H-2, H-5	H-2, H-5
7	36.9	H-7	2.80	ddt, J_1 = 11.3, J_2 = 7.2, J_3 = 4.1 Hz, 2H	H-8a, H-8b	H-6, H-2, H-8a, H-8b
8	72.8	H-8a	4.00	m, 1H	H-7, H-8b	H-1″, H-7
		H-8b	3.72	m, 1H	H-7, H-8a	H-1″, H-7
**Feruloyl group**						
1′	127.6	-	-	-	-	H-5′, H-7′, H-8′
2′	112.4	H-2′	7.21	d, J = 1.8 Hz, 1H	H-6′	
3′	146.1	-	-	-	-	H-2′, H-5′
4′	149.8	-	-	-	-	H-2′, H-5′, H-6′, H-10′
5′	116.6	H-5′	6.81	d, J = 8.0 Hz, 1H	H-6′	H-6′
6′	124.8	H-6′	7.09	dd, J_1 = 8.0, J_2 = 2.0 Hz, 1H	H-5′	H-2′, H-7′
7′	148.2	H-7′	7.65	d, J = 16.0 Hz, 1H	H-8′	H-2′, H-6′
8′	115.1	H-8′	6.37	d, J = 16.0 Hz, 1H	H-7′	H-6′, H-7′
9′	168.2	-	-	-	-	H-7′, H-8′, H-4″
10′	56.7	H-10′	3.89	s, 3H	H-2′	-
***β*-D-Glucose**						
1″	104.3	H-1″	4.37	d, J = 7.9 Hz, 1H	H-2″	H-8a, H-5″, H-2″
2″	76.2	H-2″	3.38	dd, J_1 = 9.0, J_2 = 8.0 Hz, 1H	H-1″, H-3″	H-3″
3″	81.7	H-3″	3.80	t, J = 9.0 Hz, 1H	H-2″, H-4″	H-1‴, H-2″, H-4″
4″	70.5	H-4″	4.94	t, 9.8 Hz, 1H	H-3″, H-5″	H-1‴, H-3″
5″	75.1	H-5″	3.72	d, J = 9.5 Hz, 1H	H-4″, H-6b″	H-4″
6″	68.5	H-6a″	3.75	m, 1H	H-5″, H-6b″	H-4″, H-1⁗

#	†13C	HSQC	δ 1H	Multiplicity , (J Hz)	COSY	HMBC
		H-6b″	3.48	br.d, *J* = 5.2 Hz, 1H	H-5″, H-6a″	H-4″, H-1″″
			***α*-L-Rhamnose**			
1‴	103.1	H-1‴	5.19	d, *J* = 1.67 Hz, 1H	H-2‴	H-3″
2‴	72.4	H-2‴	3.90	m, 1H	H-1‴, H-3‴	-
3‴	72.3	H-3‴	3.55	m, 1H	H-2‴, H-4‴	-
4‴	73.8	H-4‴	3.28	dd, J_1 = 9.1, J_2 = 1.4 Hz, 1H	H-3‴, H-5‴	H-5‴, H-6‴
5‴	70.9	H-5‴	3.56	* overlap	H-4‴, H-6‴	H-1‴, H-6‴, H-3″
6‴	18.5	H-6‴	1.09	d, *J* = 6.0 Hz, 3H	H-5‴	H-4‴
			***β*-D-Apiose**			
1″″	111.1	H-1″″	4.90	d, *J* = 2.2 Hz, 1H	H-2″″	H-2″″, H-4b″″,<br>H-6a″ ,H-6b″
2″″	78.1	H-2″″	3.86	d, *J* = 2.1 Hz, 1H	H-1″″	H-5″″, H-4b″″
3″″	80.6	-	-	-	-	H-4b″″
4″″	75.1	H-4a″″	3.92	d, *J* = 9.4 Hz, 1H	H-4b″″, H-5″″	H-5″″
		H-4b″″	3.73	d, *J* = 9.5 1H	H-4a″″, H-5″″	H-5″″
5″″	65.7	H-5″″	3.53	brs, 2H	H-4a″″, H-4b″″	H-4a″″, H-2″″

Table S8: NMR spectroscopic data of metabolite **10** (echinacoside), (CD_3OD, 500 MHz).

#	^{13}C	HSQC	DEPT-135	δ ^1H	Multiplicity, (*J*, Hz)	COSY	NOESY	HMBC
**Phenethyl group**								
1	131.5	-	C	-	-	-	-	H-5, H-7, H-8
2	117.2	H-2	CH	6.70	d, J = 2.0 Hz, 1H	H-6	H-7	H-6, H-7
3	146.1	-	C	-	-	-	-	H-5
4	144.7	-	C	-	-	-	-	H-2, H-6
5	116.5	H-5	CH	6.67	d, J = 8.0 Hz, 1H	H-6	-	H-6, H-7
6	121.3	H-6	CH	6.57	dd, J_1 = 8.0, J_2 = 2.0 Hz, 1H	H-2	-	H-2, H-7
7	36.6	H-7	CH_2	2.79	dt J = 7.2, J_2 = 2.5 Hz, 1H	H-8a, H-8b	H-2, H-6, H-8a, H-8b	H-2, H-6, H-8a, H-8b
8	72.0	H-8a	CH_2	4.03	m, 1H	H-7, H-8b	H-8	H-7, H-1″
		H-8b		3.73	m, 1H	H-7, H-8a	H-7	
**Caffeoyl group**								
1′	127.6	-	C	-	-	-	-	H-5′, H-7′
2′	115.2	H-2′	CH	7.06	d, J = 2.0 Hz, 1H	H-6′	-	H-6′, H-8′
3′	146.9	-	C	-	-	-	-	H-2′, H-5′
4′	149.9	-	C	-	-	-	-	H-2′, H-5′, H-6′
5′	116.3	H-5′	CH	6.77	d, J = 8.0 Hz, 1H	H-6′	-	H-6′

#	^{13}C	HSQC	DEPT-135	δ ^{1}H	Multiplicity, (*J*, Hz)	COSY	NOESY	HMBC
6′	123.3	H-6′	CH	6.96	dd, J_1 = 8.35, J_2 = 2.1 Hz, 1H	H-2, H-5′	-	H-2′, H-8′
7′	114.7	H-7′	CH	7.60	d, J = 15.9 Hz, 1H	H-8′	H-8′	H-6′, H-8′
8′	148.3	H-8′	CH	6.27	d, J = 15.9 Hz, 1H	H-7′	H-7′	H-2′, H-6′
9′	168.5	-	C=O	-	-	-	-	H-4″, H-7′, H-8′
**Central *β*-D-Glucose**								
1″	104.3	H-1″	CH	4.39	d, J = 8.0 Hz, 1H	H-2″	H-2″	H-8a, H-8b, H-2″
2″	76.2	H-2″	CH	3.39	dd, J_1 = 9.17, J_2 = 8.0 Hz, 1H	H-1″, H-3″	-	H-3″
3″	81.7	H-3″	CH	3.80	t, J = 9.2 Hz, 1H	H-2″, H-4″	H-1‴	H-2″, H-4″, H-1‴
4″	70.5	H-4″	CH	5.00	t J = 9.6 Hz, 1H	H-3″	-	H-5″, H-3″, H-1‴
5″	74.8	H-5″	CH	3.77	dd, J_1 = 9.1, J_2 = 2.3 Hz, 1H	H-4″, H-6a″	-	H-4″, H-6b″
6″	69.4	H-6a″	CH_2	3.94	dd, J_1 = 11.5, J_2 = 2.0 Hz, 1H	H-5″, H-6b″	-	H-1⁗
		H-6b″		3.63	d, J_1 = 12.0 Hz, 1H	H-5″, H-6a″	-	
***α*-L-Rhamnose**								
1‴	103.1	H-1‴	CH	5.18	d, J = 1.7 Hz, 1H	H-2‴	H-2‴, H-3‴	H-2‴, H-3‴, H-5‴
2‴	72.4	H-2‴	CH	3.91	dd, J_1 = 3.2, J_2 = 1.7 Hz, 1H	H-1‴	H-1‴	H-1‴, H-3‴
3‴	72.4	H-3‴	CH	3.57	dd, J_1 = 9.6, J_2 = 3.2 Hz, 1H	H-4‴, H-2‴	H-5‴	H-1‴, H-2‴, H-4‴
4‴	73.8	H-4‴	CH	3.28	d, J = 9.5 Hz, 1H	H-3‴	H-6‴	H-2‴, H-3‴, H-6‴

#	^{13}C	HSQC	DEPT-135	δ ^1H	Multiplicity, (J, Hz)	COSY	NOESY	HMBC
5‴	70.6	H-5‴	CH	3.56	dd, J_1 = 9.6, J_2 = 6.2 Hz, 1H	H-4‴, H-6‴	H-6‴	H-1‴, H-4‴, H-6‴
6‴	18.4	H-6‴	CH_3	1.08	d, J = 6.0 Hz, 3H	H-5‴	H-4‴, H-5‴	H-4‴
**Final *β*-D-Glucose**								
1⁗	104.7	H-1⁗	CH	4.29	d, J = 7.8 Hz, 1H	H-2⁗	H-2⁗ H-6a″, H-6b″	H-6a″, H-6b″, H-2⁗
2⁗	75.1	H-2⁗	CH	3.20	dd, J_1 = 9.1, J_2 = 7.8 Hz, 1H	H-1⁗	H-1⁗	H-6b″, H-3⁗
3⁗	77.8	H-3⁗	CH	3.22	t, J = 16.1 Hz, 1H	n.o. *overlap	*overlap	H-2⁗
4⁗	71.5	H-4⁗	CH	3.26	m, 1H	n.o. overlap	overlap	H-3⁗
5⁗	77.9	H-5⁗	CH	3.34	m, 1H	n.o. overlap	overlap	H-4⁗
6⁗	62.6	H-6a⁗	CH_2	3.82	m, 1H	H-6b⁗	H-6b⁗	H-4⁗
		H-6b⁗		3.65	d, J = 5.2 Hz, 1H	H-6a⁗	H-6a⁗	

Table S9: NMR spectroscopic data of metabolite **13** (chlorogenic acid), (CD_3OD, 500 MHz).

#	^{13}C	DEPT 135	HSQC	δ ^1H	Multiplicity, (J, Hz)	COSY	HMBC
**Quinic group**							
1	77.8	C	-	-	-	-	-
2	39.1	CH_2	$H\text{-}2_{eq}$	1.99	dd, J_1 = 15.0, J_2 = 3.2 Hz, 1H	H-3, H-5, H-2ax	-
			$H\text{-}2_{ax}$	2.10	m, 1H	H-3, H-5, H-2eq	
3	73.1	CH	H-3	4.15	br.s, 1H	H-2ax, H-4, H-2eq	H4, H-2ax, H-2eq
4	75.2	CH	H-4	3.69	dd, J_1 = 9.8, J_2 = 3.2 Hz, 1H	H-3, H-5	H-2ax, H-2eq, H5, H-6ax, H-6eq
5	72.6	CH	H-5	5.37	td, J_1 = 15.0, J_2 = 10.0, J_3 = 5.0 Hz, 1H	H-4, H-6ax, H-6eq	H-4, H-6
6	40.6	CH_2	$H\text{-}6_{eq}$	2.15	dd, J_1 = 14.8, J_2 = 3.0 Hz, 1H	H-6ax, H-5	-
			$H\text{-}6_{ax}$	2.01	d, J = 12.0 Hz, 1H	H-6eq, H-5	
7	180.8	C=O	-	-	-	-	-
**Caffeoyl group**							
1′	127.8	C	-	-	-	-	H-5′, H-7′, H-8′
2′	115.1	CH	H-2′	7.04	d, J = 2.0 Hz, 1H	H-6′	H-6′, H-7′
3′	146.8	C	-	-	-	-	-
4′	149.5	C	-	-	-	-	H-2′, H-5′, H-6′
5′	116.5	CH	H-5′	6.76	d, J = 8.3 Hz, 1H	H-6′	H-6′

6′	122.9	CH	H-6′	6.93	dd, J_1 = 8.3, J_2 = 2.0 Hz, 1H	H-5′, H-7′	H-2′, H-7′
7′	146.9	CH	H-7′	7.56	d, J = 16.0 Hz, 1H	H-8′	H-2′, H-5′, H-6′
8′	115.5	CH	H-8′	6.29	d, J = 16.0 Hz, 1H	H-7′	H-6′, H-7′
9′	169.1	C=O	-	-	-	-	H-5, H-7′, H-8′

Table S10: NMR spectroscopic data of metabolite **14** (chlorogenic acid methyl ester), (CD_3OD, 500 MHz).

#	^{13}C	DEPT 135	HSQC	H	Multiplicity, (*J*, Hz)	COSY	HMBC
**Quinic group**							
1	75.9	C	-	-	-	-	H-2, H-5, H-6
2	38.1	CH2	H-2a	2.20	m, 1H	H-3	H-4
			H-2b	2.21	dd, J_1 = 13.7, J_2 = 3.8 Hz, 1H		
3	70.6	CH	H-3	4.30	dt, J_1= 6.5, J_2 = 4.8 Hz, 1H	H-2	H-6a
4	72.4	CH	H-4	3.73	dd, J_1 = 7.5, J_2 = 3.1 Hz, 1H	H-3, H-5	H-5
5	72.1	CH	H-5	5.28	td, J_1 = 12.3, J_2 = 7.8, J_3 = 4.2 Hz, 1H	H-4	H-4
6	38.9	CH2	H-6a	2.01	dd, J_1 = 12.8, J_2 = 6.4 Hz, 1H	H-5	-
			H-6b	2.13	dd, J_1 = 13.2, J_2 = 7.9 Hz, 1H		-
7	175.5	C=O	-	-	-	-	-
8	53.1	OCH_3	H-8	3.68	s, 3H	-	-
**Caffeoyl group**							
1′	127.7	C		-	-	-	H-5′, H-7′, H-8′
2′	115.1	CH	H-2′	7.05	d, *J* = 2.0 Hz, 1H	H-6′	H-6′, H-7′
3′	146.8	C	-		-	-	H-2′, H-5′, H-6′
4′	149.5	C	-		-	-	H-2′, H-5’, H-6’
5′	116.5	CH	H-5′	6.79	d, *J* = 8.2 Hz, 1H	H-6′	-
6′	123.0	CH	H-6′	6.95	dd, J_1 = 8.2, J_2 = 2.0 Hz, 1H	H-2′, H-5′	H-2′, H-5′, H-7′
7′	147.3	CH	H-7′	7.52	d, *J* = 16.0 Hz, 1H	H-8′	H-6′
8′	115.2	CH	H-8′	6.22	d, *J* = 16.0 Hz, 1H	H-7′	H-7′, H-6′
9′	168.4	C=O	-	-	-	-	H-5, H-7′

Table S11: NMR spectroscopic data of metabolite **16** (p-hydroxybenzoic acid), (CD_3OD, 500 MHz).

#	^{13}C	HSQC	δ^1H	Multiplicity, (*J*, Hz)	COSY	HMBC
1	123.1	-	-	-	-	H3, H5
2/6	133.0	H-2, H-6	7.87	d, *J* = 8.7 Hz, 2H	H-3, H-5	-

3/5	116.0	H-3, H-5	6.80	d, J = 8.7 Hz, 2H	H-2, H-6	-
4	163.2	-	-	-	-	H2, H6
7	170.4	-	-	-	-	H2, H6

Table S12: NMR spectroscopic data of metabolite **19** (naringenin), (CD_3OD, 500 MHz).

#	^{13}C	δ ^{1}H	Multiplicity, J (Hz)
2	80.5	5.32	dd, J_1 = 12.9, J_2 = 3.0 Hz,1H
3_{ax}	44.1	3.10	dd, J_1 = 17.1, J_2 = 12.9 Hz, 1H
3_{eq}		2.68	dd, J_1 = 17.1, J_2 = 3.0 Hz,1H
4	197.7	-	-
5	168.6	-	-
6	97.1	5.89	d, J = 2.1 Hz, 1H
7	164.9	-	-
8	96.2	5.87	d, J = 2.1 Hz, 1H
9	165.5	-	-
10	103.3	-	-
1′	131.1	-	-
2′/6′	129.0	7.31	d, J = 8.4 Hz, 2H
3′/5′	116.3	6.81	d, J = 8.4 Hz, 2H
4′	159.0	-	-

Table S13: NMR spectroscopic data of metabolite **21** (hesperetin), (CD_3OD, 500 MHz).

#	^{13}C	HSQC	δ ^{1}H	Multiplicity, (J, Hz)	HMBC
2	80.3	H-2	5.31	dd, J_1 = 12.7, J_2 = 3.0 Hz, 1H	H-3_{eq}, H-2′, H-6′
3	44.1	H-3_{ax}	2.71	dd, J_1 = 17.1, J_2 = 3.0 Hz, 1H	H-2
		H-3_{eq}	3.06	dd, J_1 = 17.1, J_2 = 12.7 Hz, 1H	
4	197.6	-	-	-	H-2, H-3_{ax}, H-3_{eq}, H-8
5	164.8	-	-	-	H-8
6	97.1	H-6	5.88	d, J = 2.3 Hz, 1H	H-8
7	168.4	-	-	-	H-6, H-8

8	96.2	H-8	5.90	d, J = 2.3 Hz, 1H	H-6
9	165.5	-	-	-	H-6
10	103.4	-	-	-	H-3$_{ax}$, H-6, H-8
1′	133.2	-	-	-	H-3$_{ax}$, H-3$_{eq}$, H-2′
2′	114.6	H-2′	6.95	d, J = 2.0 Hz, 1H	H-2, H-2′, H-6′
3′	147.8	-	-	-	H-5′
4′	149.4	-	-	-	H-2′, H-6′, 4′-OCH$_3$
5′	112.6	H-5′	6.93	d, J_1= 8.3 Hz, 1H	H-6′, 4′-OCH$_3$
6′	119.3	H-6′	6.91	dd, J_1 = 8.3, J_2 = 2.0 Hz, 1H	H-2, H-5′, H-2′
4′-OCH$_3$	56.5	4′-OCH$_3$	3.86	s, 3H	-

Table S14: NMR spectroscopic data of metabolite **22** (eriodyctiol-7-O-β-D-glucoside), (CD_3OD, 500 MHz).

#	^{13}C	HSQC	δ ^{1}H	Multiplicity, (J, Hz)	COSY	HMBC
**Flavone group**						
2	80.7	H-2	5.33	dt, J_1 = 12.8, J_2 = 3.2 Hz, 1H	H-3$_{ax}$, H-3$_{eq}$	H-3$_{ax}$, H-2′,H-6′
3	44.1	H-3$_{ax}$	3.13	dd, J_1 = 17.3, J_2 = 12.8 Hz, 1H	H-3$_{eq}$	-
		H-3$_{eq}$	2.75	dd, J_1 = 17.3, J_2 = 3.1 Hz, 1H		
4	198.8	-	-	-	-	H-2, H-3$_{ax}$, H-3$_{eq}$
5	164.6	-	-	-	-	H-6, H-8
6	98.0	H-6	6.18	dd, J_1 = 2.2, J_2 = 1.3 Hz, 1H	-	H-8
7	167.0	-	-	-	-	H-1″
8	97.0	H-8	6.21	dd, J_1 = 2.2, J_2 = 1.3 Hz, 1H	-	H-6
9	n.o.	-	-	-	-	-
10	101.2	-	-	-	-	-
1′	131.7	-	-	-	-	H-2, H-6′
2′	114.8	H-2′	6.92	d, J = 1.7 Hz, 1H	H-6′	H-2, H-6′
3′	146.6	-	-	-	-	H-2′, H-5′
4′	147.0	-	-	-	-	H-2′
5′	116.3	H-5′	6.78	d, J = 8.0 Hz, 1H	H-6′	-
6′	119.3	H-6′	6.80	dd, J_1 = 8.0, J_2 = 1.7 Hz 1H	H-2′, H-5′	H-2, H-2′

***β-D*-Glucose**						
1″	101.2	H-1″	4.97	d, *J* = 7.3 Hz, 1H	H-2″	H-2″
2″	74.7	H-2″	3.44	dd, J_1 =7.8, J_2 = 6.2 Hz, 1H	H-1″	H-5″
3″	77.8	H-3″	3.40	m, 1H	H-4″	H-2″, H-4″
4″	71.2	H-4″	3.39	dd, J_1 = 9.6, J_2 = 7.3 Hz, 1H	H-3″	H-5″
5″	78.3	H-5″	3.46	m, 1H	H-6a″, H-6b″	H-2″, H-4″
6″	62.4	H-6a″	3.88	dt, J_1 = 12.2, J_2 = 2.2 Hz, 1H	H-5″, H-6b″	H-4″
		H-6b″	3.69	dd, J_1 = 12.2, J_2 = 3.2 Hz, 1H	H-5″, H-6a″	

Table S15: NMR spectroscopic data of metabolite **23** (quercetin), (CD_3OD, 500 MHz).

#	^{13}C	HSQC	DEPT-135	*δ* ^{1}H	Multiplicity, (*J*, Hz)	COSY	HMBC
2	148.1	-	C	-	-	-	H-2′, H-6′
3	137.3	-	C	-	-	-	-
4	177.4	-	C	-	-	-	-
5	162.5	-	C	-	-	-	H-6
6	99.3	H-6	CH	6.18	d, *J* = 2.1 Hz	H-8	H-8
7	165.6	-	C	-	-	-	H-6, H-8
8	94.4	H-8	CH	6.39	d, *J* = 2.1 Hz	H-6	H-6
9	158.2	-	C	-	-	-	H-8
10	104.5	-	C	-	-	-	H-6, H-8
1′	124.1	-	C	-	-	-	H-5′
2′	116.0	H-2′	CH	7.73	d, *J* = 2.1 Hz	H-6′	H-6′, H-5′
3′	146.2	-	C	-	-	-	H-2′, H-5′
4′	148.8	-	C	-	-	-	H-2′, H-6′, H-5′
5′	116.2	H-5′	CH	6.88	d, *J* = 8.7 Hz	H-6′	H-6′
6′	121.7	H-6′	CH	7.63	dd, J_1 = 8.7, J_2 = 2.1 Hz	H-5′	H-2′

Table S16: NMR spectroscopic data of metabolite **24** (isoquercetin), (CD_3OD, 500 MHz).

#	^{13}C	HSQC	*δ*^{1}H	Multiplicity, (*J*, Hz)	COSY	HMBC
**Flavonol group**						

#	^{13}C	HSQC	δ^1H	Multiplicity, (*J*, Hz)	COSY	HMBC
2	159.0	-	-	-	-	-
3	135.6	-	-	-	-	H-1″
4	179.5	-	-	-	-	-
5	163.1	-	-	-	-	-
6	99.9	H-6	6.20	d, J = 2.0 Hz, 1H	H-8	H-8
7	166.2	-	-	-	-	-
8	94.7	H-8	6.39	d, J = 2.0 Hz, 1H	H-6	H-6
9	158.5	-	-	-	-	-
10	105.7	-	-	-	-	-
1′	123.2	-	-	-	-	-
2′	117.5	H-2′	7.70	d, J = 2.2 Hz, 1H	H-6′	H-6′
3′	145.9	-	-	-	-	-
4′	149.9	-	-	-	-	-
5′	116.0	H-5′	6.87	d, J = 8.5 Hz, 1H	H-6′	H-6′
6′	123.1	H-6′	7.59	dd, J_1 = 8.5, J_2 = 2.2 Hz, 1H	H-5′	H-2′,H-5′
				***β*-D-Glucose**		
1″	104.3	H-1″	5.25	d, J = 7.5 Hz, 1H	H-2″	H-2″
2″	75.7	H-2″	3.48	dd, J_1 = 9.2, J_2 = 7.5 Hz, 1H	H-1″	H-3″
3″	78.1	H-3″	3.42	t, J = 8.8 Hz, 1H	H-2″, H-4″	H-2″, H-4″
4″	71.2	H-4″	3.34	t, J = 8.8 Hz, 1H	H-3″, H-5″	H-3″
5″	78.5	H-5″	3.21	ddd J_1 = 9.5, J_2 = 5.4, J_3 = 2.4 Hz, 1H	H-6b″	H-2″, H-4″
6″	62.6	H-6a″	3.71	dd, J_1 = 11.9, J_2 = 2.4 Hz, 1H	H-5″, H-6b″	H-4″

Table S17: NMR spectroscopic data of metabolite **25** (luteolin), (CD_3OD, 500 MHz).

#	^{13}C	δ ^1H	Multiplicity, (*J*, Hz)
2	166.6	-	-
3	103.8	6.53	s, 1H
4	183.8	-	-
5	163.2	-	-
6	100.3	6.20	d, J = 2.0 Hz, 1H

7	166.3	-	-
8	95.1	6.43	d, *J* = 2.0 Hz, 1H
9	159.4	-	-
10	105.1	-	-
1′	120.3	-	-
2′	114.1	7.37	d, *J* = 2.5 Hz,, 1H
3′	147.1	-	-
4′	151.1	-	-
5′	116.8	6.90	d, *J* = 8.5 Hz,, 1H
6′	123.6	7.39	dd, J_1 = 8.5, J_2 = 2.5 Hz,, 1H

Table S18: NMR spectroscopic data of metabolite **26** (luteolin-7-O-*β*-D-glucoside), (CD_3OD, 500 MHz).

#	^{13}C	HSQC	δ^1H	Multiplicity, (*J*, Hz)	COSY	HMBC
**Flavone group**						
2	167.2	-	-	-	-	H-3
3	103.8	H-3	6.59	s, 1H	-	-
4	184.1	-	-	-	-	H-3
5	164.8	-	-	-	-	H-6, H-8
6	101.1	H-6	6.50	d, *J* = 2.1 Hz, 1H	H-8	H-8
7	164.8	-	-	-	-	H-1″
8	96.1	H-8	6.80	d, *J* = 2.1 Hz, 1H	H-6	H-6
9	159.0	-	-	-	-	H-8
10	107.1	-	-	-	-	H-8, H,6, H-3
1′	123.1	-		-	-	-
2′	113.9	H-2′	7.39	d, *J* = 2.35 Hz, 1H	H-6′	H-6′
3′	147.8	-	-	-	-	H-5′
4′	152.1	-	-	-	-	H-2′, H-6′
5′	116.9	H-5′	6.88	d, *J* = 8.3 Hz, 1H	H-6′	-
6′	120.7	H-6′	7.41	dd, J_1= 8.3, J_2=2.3 Hz, 1H	H-2′, H-5′	H-2′
***β*-D-Glucose**						

1″	101.8	H-1″	5.07	d, J = 7.5 Hz, 1H	H-2″	H-2″
2″	74.7	H-2″	3.49	t, J=3.5 Hz, 1H	H-1″	H-4″
3″	71.3	H-3″	3.41	m, 1H	H-2″, H-4″	-
4″	78.5	H-4″	3.50	m, (overlap), 1H	H-3″	-
5″	77.9	H-5″	3.56	m, 1H	H-4″, H-6b″	H-3″, H-4″
6″	62.5	H-6a″	3.93	dd, J_1 = 12.3, J_2 = 2.3 Hz, 1H	H-5″, H-6b″	-
		H-6b″	3.72	dd, J_1 = 12.3, J_2 = 5.8 Hz, 1H	H-5″, H-6a″	

Table S19: NMR spectroscopic data of metabolite **27** (apigenin), (CD_3OD, 500 MHz).

#	^{13}C	HSQC	δ ^1H	Multiplicity, (J, Hz)	COSY	HMBC
2	166.3	-		-	-	H-3, H-2′/H-6′
3	103.8	H-3	6.60	s, 1H	-	-
4	183.9					H-3
5	163.2					H-6
6	100.2	H-6	6.21	d, J = 2.1 Hz, 1H	H-8, H-3	H-8
7	166.1					H-2′/H-6, H-3, H-8
8	95.1	H-8	6.46	d, J = 2.1 Hz, 1H	H-6, H-3	H-6
9	159.4	-		-	-	H-8
10	105.3	-		-	-	H-3, H-6, H-8
1′	123.3	-		-	-	H-3, H-3′/H-5′
2′/6′	129.5	H-2′/H-6′	7.84	d, J = 8.7 Hz, 2H	H-3′/H-5′	H-2′/H-6′
3′/5′	117.0	H-3′/H-5′	6.93	d, J = 8.7 Hz, 2H	H-2′/H-6′	H-3′/H-5′
4′	162.8	-		-	-	H-2′/H-6, H-3′/H-5′

Table S20: NMR spectroscopic data of metabolite **28** (apigenin-7-O-β-D-glucoside), (CD_3OD, 500 MHz).

#	^{13}C	HSQC	δ ^1H	Multiplicity, (J, Hz)	COSY	HMBC
**Flavone group**						
2	166.8	-	-	-	H-2′, H-6′	-
3	104.0	H-3	6.67	s, 1H	-	-

#	^{13}C	HSQC	δ ^{1}H	Multiplicity, (*J*, Hz)	COSY	HMBC
4	184.1	-	-	-	-	-
5	159.0	-	-	-	-	-
6	101.2	H-6	6.50	d, *J* = 2.2 Hz, 1H	H-8	-
7	164.9†	-	-	-	-	H-6
8	96.2†	H-8	6.83	d, *J* = 2.2 Hz, 1H	H-6	-
9	162.8	-	-	-	-	H-6
10	107.1	-	-	-	-	H-6, H-3
1′	123.1	-	-	-	-	H-3, H-3′, H-5′
2′/ 6′	129.7	H-2′, H-6′	7.89	d, *J* = 8.7 Hz, 2H	H-3′, H-5′	-
3′/ 5′	117.2	H-3′, H-5′	6.93	d, *J* = 8.7 Hz, 2H	H-2′, H-6′	-
4′	162.9⁑	-	-	-	-	H-2′, H-6′, H-3′, H-5′
***β*-D-Glucose**						
1″	101.6	H-1″	5.07	d, *J* = 7.5 Hz, 1H	H-2″, H-3″	-
2″	74.7		3.50	m, 1H	*	-
3″	77.9		3.42	m, 1H	*	-
4″	71.3		3.49	m, (overlap), 1H	*	-
5″	78.4		3.56	m, 1H	*	-
6″	62.6	6a″	3.71	dd, J_1 = 12.1, J_2 = 2.2 Hz, 1H	6b″	-
		6b″	3.93	dd, J_1 = 12.1, J_2 = 6.0 Hz, 1H	6a″	

*Unclear due to overlapping; ⁑ The correlation was done by HSQC, HMBC.

Table S21: NMR spectroscopic data of metabolite **29** (diosmetin-7-O-*β*-D-glucoside), (CD_3OD, 500 MHz).

#	^{13}C	HSQC	δ^{1}H	Multiplicity, (*J*, Hz)	COSY	HMBC
**Flavone group**						
2	164.8	-	-	-	-	H-3, H-2′, H-6′
3	104.5	H-3	6.70	s, 1H	-	-
4	184.4	-	-	-	-	-
5	159.2	-	-	-	-	H-8

#	^{13}C	HSQC	δ^1H	Multiplicity, (*J*, Hz)	COSY	HMBC
6	101.3	6	6.50	d, J = 2.3 Hz, 1H	H-6	H-8
7	168.8	-	-	-	-	-
8	96.2⁑	8	6.86	d, J = 2.3 Hz, 1H	H-8	H-6
9	156.7⁑	-	-	-	-	H-8
10	104.5	-	-	-	-	-
1′	123.4	-	-	-	-	H-2′
2′	110.9	2′	7.52	d, J = 2.0 Hz, 1H	H-6′	H-6′
3′	147.8	-	-	-	-	-
4′	150.9⁑	-	-	-	-	H-2′, H-5′, H-4′ OCH_3
5′	116.9	-	6.94	d, J = 8.3 Hz, 1H	H-6′	-
6′	122.1	6′	7.56	dd, J_1 = 8.3, J_2 = 2.0 Hz, 1H	H-2′, H-5′	H-2′
4′ -OCH_3	56.7	H-4′ OCH_3	3.97	s, 3H	-	-
***β*-D-Glucose**						
1″	101.6⁑	1″	5.08	br.d J = 7.5 Hz, 1H	H-2″	-
2″	74.8	2″	3.50	t, J = 3.5 Hz, 1H	H-1″	H-4″
3″	71.3	3″	3.40	m, 1H	H-2″	-
4″	78.4	4″	3.49	m, (overlap), 1H	H-3″, H-5″	H-2″
5″	77.9	5″	3.56	m, 1H	H-4″ , H-6a″	H-4″, H-3″
6″	62.5	6a″	3.71	dd, J_1 = 12.2, J_2 = 6.0 Hz, 1H	H-5″, H-6b″	-
		6b″	3.93	dd, J_1 = 12.2, J_2 = 2.2 Hz, 1H	H-6a″	-

⁑The correlation was done by HSQC, HMBC.

Table S22: NMR spectroscopic data of metabolite **30** (docos-1-ene), ($CDCl_3$ 500 MHz)

#	^{13}C	HSQC	DEPT 135	δ^1H	Multiplicity, (*J*, Hz)	COSY	HMBC
1	114.0	H-1a	CH_2	4.99	ddd, J_1 = 17.0, J_2 = 3.5, J_3 = 1.5 Hz, 1H	H-2	H-3
		H-1b		4.92	ddd, J_1= 10.0, J_2 = 3.5, J_3 = 1.5 Hz, 1H	H-2	
2	139.2	H-2	CH	5.81	ddt, J_1 = 17.0, J_2 = 10.0, J_3 = 5.0 Hz, 1H	H-3, H-1a, H1b	H-3

3	33.8	H-3	CH_2	2.04	tdd, J_1 = 14.5, J_2 = 8.0, J_3 = 1.5 Hz, 2H	H-2	H-1a, H-1b, H-2
4-18	29.7	H-4/18	$(CH_2)_{15}$	1.25	br.s. 30H	-	-
19	29.1	H-19	CH_2	1.37	q, J = 7.1 Hz, 2H	-	-
20	31.9	H-20	CH_2	1.29	m, 2H	-	H-21, H-22
21	22.6	H-21	CH_2	1.31	br. q, J = 7.0 Hz, 2H	H-22	H-22
22	14.1	H-22	CH_3	0.88	t, J = 7.0 Hz, 3H	H-21	H-21

Note S1: NMR data of metabolite **11**, (CD_3OD, 500 MHz)

p-coumaric acid: ^{1}H NMR (CD_3OD, 500 MHz) δ: 7.52 (d, J = 16.0 Hz, 1H, H-7), 7.42 (d, J = 8.7 Hz, 2H, H-2/6), 6.80 (d, J = 8.7 Hz, 2H, H-3/5), 6.29 (d, J = 16.0 Hz, 1H, H-8). ^{13}C-NMR (500 MHz, CD_3OD) δ: 171.1 (C-9), 161.2 (C-4), 146.6 (C-7), 131.1 (C-2, 6), 127.3 (C-1), 116.8 (C-3, 5), 115.7 (C-8).

Note S2: NMR data of metabolite **12**, (CD_3OD, 500 MHz)

Caffeic acid: ^{1}H-NMR (CD_3OD, 500 MHz) δ 7.02 (d, J = 2.0 Hz, 1H, H-2), 6.76 (d, J = 8.0 Hz, 1H, H-5), 6.91 (dd, J_1 = 8.0, J_2 = 2.0 Hz, 1H, H-6), 7.51 (d, J = 16.0 Hz, 1H, H-7), 6.23 (d, J = 16.0Hz, 1H, H-8).

Note S3: NMR data of metabolite **15**, (CD_3OD, 500 MHz)

5,7-dihydroxychromone: ^{1}H NMR (CD_3OD, 500 MHz), δ: 7.96 (d, J = 6.0 Hz, 1H, H-2), 6.18 (d, J = 6.0 Hz, 1H, H-3), 6.19 (d, J = 2.0 Hz, 1H, H-6), 6.32 (d, J = 2.0 Hz, 1H, H-8).

Note S4: NMR data of metabolite **17**, (CD_3OD, 500 MHz)

Gallic acid: ^{1}H NMR (CD_3OD, 500 MHz), δ: 7.1 (s, 2H, H-2 / H-6). ^{13}C NMR (500 MHz, CD_3OD), δ: 171.2 (C-7) 146.4 (C-3 / C-5), 139.4 (C-4), 123.1 (C-1) 110.3 (C-2 / C-6).

Note S5: NMR data of metabolite **18**, (CD_3OD, 500 MHz)

Vanilic acid :^{1}H-NMR (CD_3OD, 500 MHz) δ 7.54 (d, J = 2.0 Hz, 1H, H-2), 7.55 (dd, J_1 = 8.7, J_2 = 2.0 Hz, 1H, H-6), 6.84 (d, J = 8.7 Hz, 1H, H-5), 3.89 (s, 3H). ^{13}C-NMR (500 MHz, CD_3OD): δ 170.1 (COOH), 152.7 (C-3), 148.7 (C-4), 125.3 (C-6), 123.1 (C-1), 115.9 (C-2), 113.8 (C-5), 56.4 (C-8, OCH_3).

Note S6: NMR data of metabolite **20**, (CD_3OD, 500 MHz)

Eriodyctiol: ^{1}H-NMR (CD_3OD, 500 MHz), δ: 6.91 (*br*d, J = 1.5, 1H, H-2′), 6.77 (d, J = 1.5 Hz, 1H, H-5′), 6.81 (m, 1H, H-6′) 5.89 (d, J = 2.1 Hz, 1H, H-8), 5.87 (d, J = 2.1 Hz, 1H, H-6), 5.28 (dd, J_1 = 13.0, J_2 = 3.0 Hz, 1H, H-2), 3.06 (dd, J_1 = 17.2, J_2 = 12.8 Hz, 1H, H_{ax}-3), 2.69 (dd, J_1=17.2, J_2 = 3.1 Hz, 1H, H_{eq}-3).
